# APP‐C31: An Intracellular Promoter of Both Metal‐Free and Metal‐Bound Amyloid‐β_40_ Aggregation and Toxicity in Alzheimer's Disease

**DOI:** 10.1002/advs.202307182

**Published:** 2023-11-10

**Authors:** Eunju Nam, Yuxi Lin, Jiyong Park, Hyunsu Do, Jiyeon Han, Bohyeon Jeong, Subin Park, Da Yong Lee, Mingeun Kim, Jinju Han, Mu‐Hyun Baik, Young‐Ho Lee, Mi Hee Lim

**Affiliations:** ^1^ Department of Chemistry Korea Advanced Institute of Science and Technology (KAIST) Daejeon 34141 Republic of Korea; ^2^ Research Center for Bioconvergence Analysis Korea Basic Science Institute (KBSI) Ochang Chungbuk 28119 Republic of Korea; ^3^ Center for Catalytic Hydrocarbon Functionalizations Institute for Basic Science (IBS) Daejeon 34141 Republic of Korea; ^4^ Graduate School of Medical Science and Engineering KAIST Daejeon 34141 Republic of Korea; ^5^ Rare Disease Research Center Korea Research Institute of Bioscience and Biotechnology (KRIBB) Daejeon 34141 Republic of Korea; ^6^ Department of Biochemistry Department of Medical Science Chungnam National University School of Medicine Daejeon 35015 Republic of Korea; ^7^ Bio‐Analytical Science University of Science and Technology (UST) Daejeon 34113 Republic of Korea; ^8^ Graduate School of Analytical Science and Technology Chungnam National University Daejeon 34134 Republic of Korea; ^9^ Department of Systems Biotechnology Chung‐Ang University Gyeonggi 17546 Republic of Korea; ^10^ Frontier Research Institute for Interdisciplinary Sciences Tohoku University Miyagi 980‐8578 Japan

**Keywords:** accelerator toward amyloidogenesis, amyloid precursor protein, amyloid‐β, metal ions, protein–protein interaction

## Abstract

Intracellular *C*‐terminal cleavage of the amyloid precursor protein (APP) is elevated in the brains of Alzheimer's disease (AD) patients and produces a peptide labeled APP‐C31 that is suspected to be involved in the pathology of AD. But details about the role of APP‐C31 in the development of the disease are not known. Here, this work reports that APP‐C31 directly interacts with the *N*‐terminal and self‐recognition regions of amyloid‐β_40_ (Aβ_40_) to form transient adducts, which facilitates the aggregation of both metal‐free and metal‐bound Aβ_40_ peptides and aggravates their toxicity. Specifically, APP‐C31 increases the perinuclear and intranuclear generation of large Aβ_40_ deposits and, consequently, damages the nucleus leading to apoptosis. The Aβ_40_‐induced degeneration of neurites and inflammation are also intensified by APP‐C31 in human neurons and murine brains. This study demonstrates a new function of APP‐C31 as an intracellular promoter of Aβ_40_ amyloidogenesis in both metal‐free and metal‐present environments, and may offer an interesting alternative target for developing treatments for AD that have not been considered thus far.

## Graphical abstract

In an Alzheimer's disease (AD)‐affected brain, the releasing of a small peptide, APP‐C31, from the amyloid precursor protein (APP) is increased. The toxicity of APP‐C31 is relatively known, but its role in the pathology of AD remains unclear. These new findings demonstrate that APP‐C31 forms transient adducts with both metal‐free and metal‐bound Aβ_40_ species and, consequently, promotes their aggregation and toxicity.

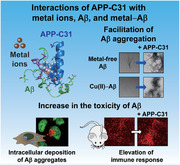

## 1 Introduction

**Figure 1:**
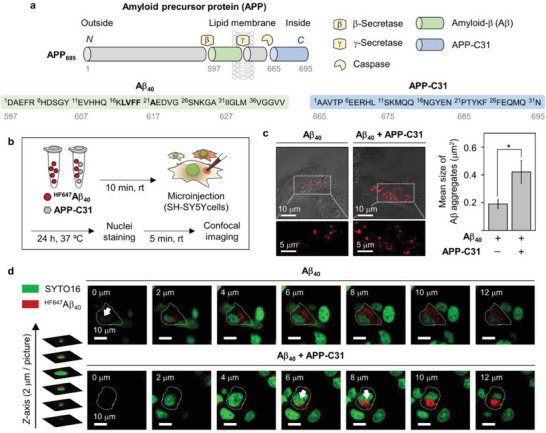
Impact of APP‐C31 on the intracellular aggregation of Aβ_40_. a) Amino acid sequences of APP‐C31 (APP_665–695_) and Aβ_40_ that are generated from the proteolytic cleavage of APP. The self‐recognition site of Aβ is highlighted in bold. b) Scheme of the microinjection experiments. c) Mean size of intracellular Aβ_40_ aggregates in the absence and presence of APP‐C31 observed by confocal microscopy. Aggregates (red) with dimensions exceeding the resolution limit were analyzed from three or more randomly selected fields per condition. Conditions: [^HF647^Aβ_40_] = 10 µm; [APP‐C31] = 10 µm; 24 h incubation. Scale bars = 5 and 10 µm. Data are represented as mean ± s.e.m. *n* = 52 for the group of ^HF647^Aβ_40_; *n* = 63 for the group of ^HF647^Aβ_40_ with APP‐C31; *p < 0.05; Student's *t*‐test. The size distribution of Aβ_40_ aggregates detected in both groups was summarized in Figure S2, Supporting Information. d) Z‐stack images of Aβ_40_ aggregates (red) with and without APP‐C31. Nuclei were stained with SYTO16 (green). ^HF647^Aβ_40_ aggregates observed from the nuclear region were indicated with white arrows. Individual green and red fluorescence images are presented in Figure S3, Supporting Information. Conditions: [^HF647^Aβ_40_] = 10 µm; [APP‐C31] = 10 µm; [SYTO16] = 2.5 µm; 24 h incubation. Scale bar = 10 µm.

The accumulation and misdistribution of amyloid precursor protein (APP) fragments are associated with the pathology of Alzheimer's disease (AD).^[^ ^1^, ^2^, ^3^, ^4^, ^5^, ^6^ ^]^ As a type‐I transmembrane protein, APP is localized in cell surfaces or membranes of organelles.^[^ ^1^, ^7^ ^]^ Cleavage of APP by enzymes such as α‐, β‐, and γ‐secretases releases various fragments that participate in neurotrophic activities, synaptic plasticity, and intracellular signaling.^[^ ^1^ ^]^ Amyloid‐β (Aβ) peptides with 38–43 amino acid residues are generated via the sequential cleavage of APP by β‐ and γ‐secretases,^[^ ^1^, ^2^, ^3^, ^4^, ^7^, ^8^ ^]^ as illustrated in **Figure** **1**a. Aβ peptides self‐assemble into amyloid oligomers and fibrils that trigger neurodegeneration observed in AD.^[^ ^1^, ^2^, ^3^, ^4^, ^7^, ^8^ ^]^ The classical amyloid cascade hypothesis suggests that extracellular Aβ aggregates result in synaptic loss and memory impairment;^[^ ^2^, ^3^ ^]^ however, emerging evidence like the progression of cognitive deficits prior to forming senile plaques leads to the concept that Aβ aggregates inside the cells may also be neurotoxic.^[^ ^4^, ^8^, ^9^, ^10^ ^]^ Under pathological conditions, it has been identified that Aβ species can be accumulated in perinuclear and nuclear regions rather than transported outside the cell through secretory pathways.^[^ ^4^, ^5^, ^9^, ^10^, ^11^ ^]^ These intracellular Aβ aggregates induce the dysfunction of organelles and neuronal death.^[^ ^4^, ^5^, ^9^, ^10^, ^11^ ^]^

As depicted in Figure 1a, a cytoplasmic peptide carrying 31 amino acid residues denoted APP‐C31 (APP_665–695_) is produced upon cleavage of the *C*‐terminal region of APP by caspases.^[^ ^1^, ^6^, ^12^, ^13^, ^14^, ^15^, ^16^, ^17^, ^18^ ^]^ In AD‐affected brains, the deposition of APP *C*‐terminal domains at hippocampal regions as well as the increase in the amounts of activated caspases and caspase‐mediated APP fragments are observed.^[^ ^3^, ^6^, ^12^, ^13^, ^14^, ^15^, ^16^, ^17^, ^18^ ^]^ Like Aβ, APP‐C31 contributes to neuronal toxicity leading to apoptosis and synaptic depression.^[^ ^6^, ^12^, ^13^, ^14^, ^15^, ^16^, ^17^, ^18^ ^]^ The role of APP‐C31 in facilitating the accumulation of Aβ_42_ has also been suggested, implicating its involvement in activating the amyloidogenic cleavage of APP and altering the Aβ_42_‐to‐Aβ_40_ ratio.^[^ ^14^ ^]^ The knockout of caspases or the mutation of APP from Asp664 to Ala whose role in inhibiting the generation of APP‐C31 reduces the formation of Aβ plaques, diminishes synaptic loss, and prevents neurodegeneration in AD transgenic mice.^[^ ^17^, ^18^, ^19^ ^]^ Thus, these findings imply that the amount of intracellular APP‐C31 is augmented and, consequently, neurotoxicity is enhanced when AD develops. Recently, the co‐localization of *C*‐terminal APP fragments with Aβ was observed in cellular regions, including autolysosomes, perinuclear compartments, and multi‐vesicular bodies, in the brains of AD transgenic mice.^[^ ^5^, ^20^, ^21^, ^22^ ^]^ Moreover, the studies with the *C*‐terminal truncated APP, APP_1–664_, and mutant APP(Asp664Ala) suggested that Aβ could activate caspases inducing the *C*‐terminal cleavage of APP generating APP‐C31.^[^ ^17^, ^18^, ^19^, ^22^ ^]^ Despite these indications, the mechanism of how APP‐C31 is involved in the pathology driven by Aβ is not known to date.

Moreover, in AD‐afflicted brains, metal ion dyshomeostasis and highly concentrated metals trapped in senile plaques such as ≈400 µm and 1 mm of copper and zinc, respectively, are found.^[^ ^7^, ^23^ ^]^ The accumulation of Aβ aggregates with metal ions is also detected in intracellular systems.^[^ ^24^, ^25^ ^]^ Metal ions, including Cu(I/II) and Zn(II), can coordinate to Aβ forming metal‐bound Aβ (metal–Aβ) and, subsequently, affect Aβ aggregation pathways to different degrees depending on the type of metal ions and the metal‐to‐Aβ stoichiometry.^[^ ^7^, ^23^, ^24^, ^25^, ^26^, ^27^, ^28^ ^]^ In this context, a better understanding of how APP‐C31 interacts with metal ions and metal–Aβ is also of significant interest.

We questioned whether APP‐C31 interacts with both metal‐free Aβ and metal–Aβ and alters their aggregation and toxicity profiles. Thus, we evaluated the effects of APP‐C31 on modifying the aggregation and toxicity of intracellular Aβ_40_ through live‐cell investigations employing microinjection methods. In addition, the influence of APP‐C31 on the aggregation kinetics and pathways of both metal‐free and metal‐bound Aβ_40_ and their detailed molecular‐level interactions were determined. Moreover, the impact of Aβ_40_ incubated with APP‐C31 on neuronal growth and inflammation was probed in human neurons and in vivo (murine brains). Overall, our work substantiates that an intracellular *C*‐terminal fragment, APP‐C31, can provoke the aggregation behavior and toxicity of both metal‐free and metal‐bound Aβ_40_ through the interactions between the peptides, which illuminates a new role of APP‐C31 as an intracellular promoter in the pathology of AD.

## 2 Results and Discussion

The intracellular environment is crowded, and 20%–30% of the volume is occupied by macromolecules.^[^ ^29^ ^]^ In these systems, effective concentrations of biomolecules are higher than their actual concentrations in the test tube due to the excluded volume effect.^[^ ^29^ ^]^ Moreover, the spatiotemporal localizations of proteins could induce their concentrations up to a millimolar level.^[^ ^30^ ^]^ Analyzing intracellular Aβ and APP‐C31 is hampered by heterogeneous intracellular systems, but it has been suggested that intracellular Aβ can be accumulated exhibiting a significantly higher concentration than extracellular Aβ.^[^ ^31^ ^]^ Given that our investigations have the detection limit at low concentrations of peptides, we considered all these aspects to set their concentrations used for individual experiments.

### 2.1 Impact of APP‐C31 on the Aggregation of Aβ

To determine whether APP‐C31 affects the aggregation of Aβ at the intracellular region, microinjection experiments, which can have the high transduction efficiency of biomolecules and the precise control of injection volume to the cytosol,^[^ ^32^ ^]^ were conducted in combination with high‐resolution confocal microscopy. It should be noted that the trafficking of peptides or proteins injected into the cytosol may not be the same as that of the peptides or proteins internalized through endosomes, but microinjection would be valuable for monitoring their aggregation behaviors under intracellular conditions.^[^ ^32^, ^33^, ^34^ ^]^ Aβ_40_ conjugated to the HiLyte Fluor 647 (HF647) fluorophore at the *N*‐terminus (^HF647^Aβ_40_) was employed to monitor intracellular Aβ aggregates in living cells due to its similar aggregation kinetics, compared to that of fluorophore‐unlabeled Aβ_40_ (Figure S1, Supporting Information), and the relatively high photostability of the HF647 fluorophore.^[^ ^35^, ^36^ ^]^ It should be noted that Aβ_42_ was not used for microinjection experiments because its rapid aggregation causes the clogging of injection tips.

The size of individual Aβ_40_ aggregates was determined by counting the pixels of the fluorescent area following previously reported procedures.^[^ ^36^, ^37^ ^]^ As described in Figure 1b, the femtoliter volume of solutions containing ^HF647^Aβ_40_ with and without APP‐C31 was injected into the cytoplasm.^[^ ^38^ ^]^ After 24 h incubation, ^HF647^Aβ_40_ in the absence of APP‐C31 exhibited a cluster of spherical aggregates with an average size of ≈0.19 µm^2^, as illustrated in Figure 1c and Figure S2, Supporting Information. When ^HF647^Aβ_40_ with APP‐C31 was introduced inside the cells, we detected enlarged Aβ_40_ aggregates with a cluster size of about 0.42 µm^2^. The intracellular distribution of ^HF647^Aβ_40_ aggregates was further monitored in the cells stained with the fluorescent SYTO16 dye that can visualize nuclei by binding to nucleic acids,^[^ ^39^ ^]^ as displayed in Figure 1d and Figure S3, Supporting Information. Aβ_40_ aggregates without APP‐C31 were mostly located in the cytoplasm and few clusters of them were internalized at the nucleus, as highlighted with white arrows (Figure 1d). When ^HF647^Aβ_40_ was injected with APP‐C31 into the cells, large Aβ_40_ aggregates invaded into the nucleus. These findings support that APP‐C31 can promote the aggregation of Aβ_40_ in intracellular regions and induce the deposition of Aβ_40_ aggregates particularly at the nuclear and perinuclear regions.

**Figure 2:**
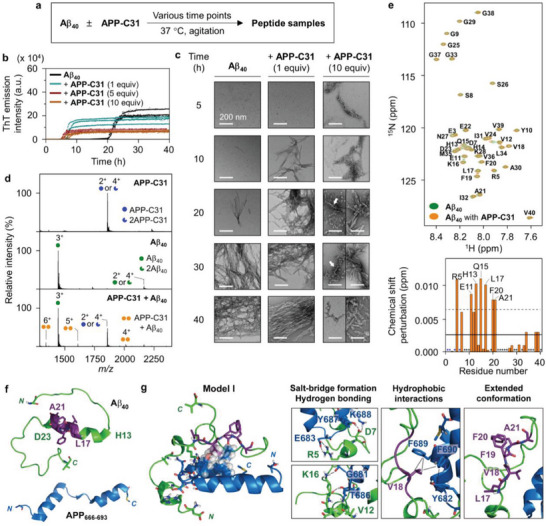
Effect of APP‐C31 on the aggregation of Aβ_40_ and its interactions with Aβ_40_. a) Scheme of the aggregation experiments. b) Degree on the aggregation of Aβ_40_ upon incubation with or without APP‐C31 analyzed by the ThT assay. Experiments were carried out in triplicate. c) Morphology of the peptide aggregates produced by treatment of Aβ_40_ with or without APP‐C31 detected by TEM. White arrows indicate a mixture of globular aggregates and short fibrils. Scale bar = 200 nm. Conditions: [Aβ_40_] = 20 µm and [APP‐C31] = 20, 100, and 200 µm; 20 mm HEPES, pH 7.4, 150 mm NaCl; 37 °C; constant agitation (559 cpm). d) ESI–MS spectra of APP‐C31, Aβ_40_, and APP‐C31 incubated with Aβ_40_. Charge states are marked in the MS spectra. Conditions: [APP‐C31] = 100 µm; [Aβ_40_] = 100 µm; 20 mm ammonium acetate, pH 7.3; 37 °C; 2 h incubation; no agitation. The samples were diluted by tenfold prior to injection to the mass spectrometer. e) Interaction of APP‐C31 with monomeric Aβ_40_ analyzed by 2D ^1^H–^15^N HSQC NMR (900 MHz). The average of CSPs and the average + one standard deviation are indicated with solid and dashed lines, respectively. The zoomed‐in images of the regions where noticeable CSPs appear are depicted in Figure S11, Supporting Information. Conditions: [^15^N‐labeled Aβ_40_] = 40 µm; [APP‐C31] = 200 µm; 20 mm HEPES, pH 7.4; 5 °C. The amino acid residues indicated in blue or black asterisks represent the residues that were unresolved or not significantly shifted, respectively. f) Conformations of Aβ_40_ (PDB 2LFM)^[^ ^55^ ^]^ and APP_666–693_ [PDB 3DXC; as a part of APP‐C31 (APP_665–695_)]^[^ ^56^ ^]^ used for metadynamics MD simulations. The self‐recognition site of Aβ shown in Figure 1a is highlighted in purple. g) A representative model of the APP_666–693_–Aβ_40_ interfaces from the trajectories of MD simulations. Possible hydrogen bonds within 3.0 Å and hydrophobic interactions observed within 4.0 Å are indicated with dashed black lines. The amino acid residues involved in hydrophobic interactions are presented in the space‐filling representation. The other representative model, Model II, is illustrated in Figure S16, Supporting Information.

Our cell studies showed the striking effects of APP‐C31 on intracellular Aβ_40_ aggregation and deposition; however, some experimental limitations existed. First, since the cell environment is heterogeneous and composed of many biomolecules, it is challenging to investigate the direct impact of APP‐C31 on the aggregation kinetics of Aβ. Second, due to the conjugation of a fluorophore to Aβ_40_, which induces differences in the physiochemical property and structural conformation of Aβ,^[^ ^35^ ^]^ APP‐C31 binding to Aβ_40_ may be affected by the presence of a fluorophore. Thus, we evaluated the effect of APP‐C31 on the aggregation kinetics of unlabeled Aβ_40_ in aqueous media by the thioflavin‐T (ThT) fluorescence assay used for quantifying the formation of amyloid fibrils.^[^ ^7^, ^40^, ^41^ ^]^ As summarized in **Figure** **2**a,b, the change in fluorescence upon incubation of Aβ_40_ showed a sigmoidal curve with a lag phase followed by an elongation phase, indicating a nucleation‐dependent fibrillar aggregation. The averaged lag time, *t*_lag_, and the averaged time to reach the half‐maximum fluorescence intensity, *t*_1/2_, were analyzed to be 19.3 (±0.2) h and 21.5 (±0.1) h, respectively. When Aβ_40_ was treated with the stoichiometric amount of APP‐C31, the lag phase was noticeably shortened exhibiting the decreased *t*_lag_ and *t*_1/2_ values [*t*_lag_ = 6.5 (±0.2) h; *t*_1/2_ = 7.3 (±0.2) h] that were similar to those under the suprastoichiometric conditions. Moreover, the maximum ThT fluorescence intensity where Aβ_40_ aggregation reached the plateau became lower as a function of APP‐C31's concentration. Therefore, these results suggest that APP‐C31 facilitates the nucleation‐dependent Aβ_40_ aggregation and produces different Aβ_40_ aggregates depending on the APP‐C31‐to‐Aβ stoichiometry.

The aggregation kinetics of Aβ_40_ with APP‐C31 was also probed in the presence of seeds generated from preformed amyloid fibrils of Aβ_40_. Aβ monomers form nuclei via primary nucleation, which is thermodynamically unfavorable, followed by the elongation of amyloid fibrils, whereas their contact onto the surface of preformed aggregates catalyzes the generation of nuclei (i.e., secondary nucleation).^[^ ^42^ ^]^ As shown in Figure S4, Supporting Information, Aβ_40_ seeds escalated the aggregation of Aβ_40_ with the *t*_lag_ and *t*_1/2_ values of 3.0 (±0.2) h and 3.5 (±0.2) h, respectively, which was not significantly altered with the addition of APP‐C31 [*t*_lag_ of 2.3 (±0.2) h; *t*_1/2_ of 3.1 (±0.2) h]. These results imply that APP‐C31 could be involved in the primary nucleation of Aβ_40_ aggregation over its seed‐dependent aggregation pathways. It should be noted that APP‐C31 itself did not show a significant change in the secondary structure and negligibly affected the ThT fluorescence (Figures S5 and S6, Supporting Information); thus, APP‐C31 itself could not generate β‐structured amyloid fibrils under these experimental conditions.

To verify the morphology of Aβ_40_ aggregates produced with different amounts of APP‐C31, the resultant peptide samples were visualized by transmission electron microscopy (TEM). The sample of Aβ_40_ only presented small and thin fibrils after 20 h incubation, as presented in Figure 2c. In the presence of APP‐C31 (1 equiv), amyloid fibrils were notably generated starting from 10 h incubation. When Aβ_40_ was incubated with 10 equiv of APP‐C31, we detected Aβ_40_ protofibrils at the early aggregation stage. In addition, curvilinear fibrils appeared from Aβ_40_ treated with 10 equiv of APP‐C31. Different from Aβ_40_ added with equimolar APP‐C31, longer incubation of these samples showed bundles of short fibrils and a mixture of globular agglomerates and branched fibrils (marked with white arrows). Some of APP‐C31 may form the globular agglomerates at its high concentration (Figure S7, Supporting Information). These observations imply that the morphological changes of Aβ_40_ aggregates may also be induced by nonfibrillar APP‐C31 aggregates, in addition to monomeric APP‐C31. In the case of Aβ_42_, its aggregation kinetics [*t*_1/2_ of 1.6 (±0.2) h] was slightly altered in the presence of APP‐C31 [*t*_1/2_ of 1.1 (±0.2) h], as shown in Figure S8, Supporting Information. Notably, the treatment of APP‐C31 varied the morphology of Aβ_42_ aggregates, as presented in Figure S8c, Supporting Information. Aβ_42_ incubated with APP‐C31 for 8 h was observed to generate long, thick, and straight Aβ fibrils while Aβ_42_ without APP‐C31 produced relatively shorter and thinner amyloid fibrils. These results suggest that APP‐C31 could affect the polymorphic amyloid formation of Aβ_42_.

To further probe the effect of Aβ on APP‐C31 aggregation, the molecular weight distribution of the resultant peptide species was monitored by gel electrophoresis/Western blot (gel/Western blot) employing two antibodies, including an anti‐Aβ antibody (6E10 for Aβ) and an anti‐APP *C*‐terminus antibody (Y188 for APP‐C31) (Figure S9, Supporting Information). When APP‐C31 was incubated for 24 h, the molecular weight of ≈4 kDa corresponding to its monomeric form was indicated by Y188. Upon incubation of APP‐C31 with Aβ_40_, a band at ≈7 kDa with faint smearing ranging from 4 to 270 kDa additionally appeared. Given that the band at ≈7 kDa with smearing was also detected by 6E10, the complexation between APP‐C31 and Aβ_40_, instead of the aggregation of APP‐C31 induced by Aβ_40_, was suggested. Taken together, our aggregation studies corroborate that APP‐C31 can accelerate the aggregation of Aβ_40_ in aqueous media as well as inside living cells.

### 2.2 Interaction of APP‐C31 with Aβ_40_

To provide mechanistic insight into how APP‐C31 modifies the aggregation profile of Aβ, we assessed the molecular‐level interaction between APP‐C31 and Aβ. Given that i) APP‐C31 showed the noticeable impact on the aggregation kinetics of Aβ_40_, compared to Aβ_42_, and ii) the rapid aggregation of Aβ_42_ could induce the disappearance of peak signals, we used Aβ_40_ for investigating the interaction between two peptides. When Aβ_40_ incubated with APP‐C31 was monitored by electrospray ionization–mass spectrometry (ESI–MS), a soft ionization method used in the characterization of protein complexes,^[^ ^43^ ^]^ new peaks at 1342, 1610, and 2012 m z^−1^ were observed with +6, +5, and +4 charge states, respectively, which represent that APP‐C31 binds to Aβ_40_ in a 1:1 ratio (Figure 2d). Binding events between APP‐C31 and Aβ_40_, detected by isothermal titration calorimetry (ITC), indicated the spontaneous reaction with Δ*G* = −5.0 (±0.1) kcal mol^−1^ and Δ*H* = −1.5 (±0.3) kcal mol^−1^, as presented in Figure S10, Supporting Information. The *T*Δ*S* term was found to be 3.5 (±0.3) kcal mol^−1^. These thermodynamic properties are curious on first sight, as a reaction in which two particles associate to form a single species is expected to be entropically disfavored. Specifically, it is the translational entropy associated with each particle amounting to roughly 10 kcal mol^−1^ at room temperature that disfavors associative processes reducing the number of free particles.^[^ ^44^ ^]^ A minor but relevant feature is the loss of structural flexibility upon aggregation, which also reduces conformational entropy. The increase in entropy observed here is likely due to the release of water molecules that were tightly bound to the protein surfaces and become free water molecules as the two peptides form an association complex. The binding affinity of APP‐C31 for Aβ_40_ was measured to be 149.7 (±11.4) µm, indicative of relatively weak protein–protein interactions.^[^ ^45^ ^]^

The interaction between APP‐C31 and Aβ_40_ in solution was supported by 2D ^1^H–^15^N heteronuclear single quantum coherence nuclear magnetic resonance (2D ^1^H–^15^N HSQC NMR) spectroscopy at the amino acid residue level. As illustrated in Figure 2e and Figure S11, Supporting Information, upon treatment of APP‐C31 with a solution of uniformly ^15^N‐labeled Aβ_40_, we detected notable chemical shift perturbations (CSPs) of the Arg5, Glu11, His13, Gln15, Leu17, Phe20, and Ala21 residues in ^15^N‐labeled Aβ_40_. This observation indicates that APP‐C31 could interact with the *N*‐terminal region and the self‐recognition site of Aβ_40_ (i.e., Leu17 to Ala21; Figure 1a). Given that the self‐recognition site of Aβ is a central hydrophobic region responsible for the oligomerization and fibrilization,^[^ ^7^, ^8^ ^]^ APP‐C31 may alter the conformation and aggregation propensity of Aβ (vide infra). In addition to monomeric Aβ, we probed the interaction between APP‐C31 and amyloid fibrils of Aβ_40_ by 1D ^1^H NMR spectroscopy. As depicted in Figure S12, Supporting Information, the peak intensities of APP‐C31 were notably diminished by ≈40% when it was incubated with Aβ_40_ fibrils, thereby supporting its potential interaction with Aβ fibrils. Collectively, the abovementioned ESI–MS, ITC, and NMR studies verify that APP‐C31 can interact with Aβ in a relatively weak binding manner.

### 2.3 Potential APP‐C31–Aβ_40_ Binding Modes

To obtain atomistic insight into the binding of APP‐C31 to Aβ_40_, a series of molecular dynamics (MD) simulations was performed.^[^ ^46^, ^47^, ^48^, ^49^, ^50^, ^51^, ^52^, ^53^ ^]^ As Aβ is an intrinsically disordered peptide, multiple structural ensembles can be generated depending on experimental conditions.^[^ ^54^, ^55^ ^]^ In these computer simulations, we sought to use a structure of monomeric Aβ_40_ observed in aqueous solutions without organic solvents and hydrophobic micelles. Thus, the structure of Aβ_40_ obtained by solution NMR (PDB 2LFM;^[^ ^55^ ^]^ Figure 2f), which meets our criteria, was employed as a starting conformation of monomeric Aβ_40_ in our simulations.^[^ ^55^ ^]^ In addition, the atomistic structure of APP‐C31 is not known; thus, the X‐ray crystal structure of APP_666–693_ excised from the intracellular domain of APP complexed with Fe65 protein (PDB 3DXC;^[^ ^56^ ^]^ Figure 2f) was selected as the starting guess structure of APP‐C31. Modelling slow processes, like the association of peptides taking place in minutes to hours of time‐scale, with MD simulations is challenging. One solution to accelerating the simulation and obtaining meaningful data within a tractable computing time is to first conduct metadynamics MD simulations that allow a more rapid and efficient exploration of the wider configurational space to identify the most relevant adduct structures. Using the multi‐walker metadynamics algorithm,^[^ ^53^ ^]^ we sampled dimeric configurations with the gross metadynamics simulation time being 8.28 µs. These simulations facilitate the conformational sampling along the center‐of‐mass (COM) coordinate between the pair of proteins, and provide the ensemble of the dimeric interface between the two proteins.^[^ ^46^ ^]^ As depicted in Figure S13a, Supporting Information, we found the most stable hetero‐dimer conformers at the COM distance of 13 Å. The observed COM distance profile shown in Figure S13c, Supporting Information, also suggests that the association of the two proteins is reversible.

To verify and rank the relative stabilities of the dimeric structures, additional 500 ns of unbiased MD simulations without the metadynamics acceleration were conducted, where the initial structures were taken from the metadynamics MD simulations. The adduct structures from the MD simulations were evaluated based on two criteria: i) the monomers of Aβ_40_ and APP_666–693_ maintain contacts throughout the course of unbiased MD simulations (Figure S14, Supporting Information); ii) seven amino acid residues displaying the pronounced CSPs are at the dimeric interface (Figure S15, Supporting Information). Figure 2g and Figure S16, Supporting Information illustrate two classes of stable APP_666–693_–Aβ_40_ dimeric adduct structures that are most consistent with our experimental results described in Figure 2e. In Model I and II, the dimeric interfaces are formed by favorable intermolecular contacts and hydrogen bonding. The amino acid residues in the *N*‐terminal region and the self‐recognition site of Aβ_40_ exhibit intermolecular ionic interactions with APP_666–693_. Both models present a salt bridge between Arg5 of Aβ_40_ and Glu683 of APP_666–693_. Other salt bridges are detected between Asp7 of Aβ_40_ and Lys688 of APP_666–693_ (Model I) or Lys16 of Aβ_40_ (Model II). These salt bridges can further neutralize the electrostatic charge in the *N*‐terminal region of Aβ_40_, as presented in Figure S17, Supporting Information, suggesting that the protein–protein interactions reduce the electrostatic repulsion that can interfere with Aβ aggregation.^[^ ^40^ ^]^ Additionally, multiple hydrogen bonds are observed in the APP_666–693_–Aβ_40_ dimeric interfaces. The Asp7 residue of Aβ_40_ constructs hydrogen bonds with Tyr687 and Asn684 of APP_666–693_ in Model I and II, respectively. Furthermore, the backbone carbonyl group of Val12 (Model I) and the imidazole nitrogen atom of His13 (Model II) in Aβ_40_ produce the backbone–side chain and side chain–side chain hydrogen bonds with Asn684 of APP_666–693_, respectively. Hydrogen bonding between Lys16 adjacent to the self‐recognition site of Aβ_40_ and the backbone carbonyl group of Gly681 or the hydroxyl group of Thr686 in APP_666–693_ is also monitored in Model I. As indicated in Model II, Thr686 of APP_666–693_ additionally forms hydrogen bonding with the backbone amide moiety of Val18 in Aβ_40_. Moreover, the intermolecular hydrophobic contacts between the aliphatic side chain of Val18 in Aβ_40_ and the aromatic rings of Tyr682, Phe689, and Phe690 in APP_666–693_ are revealed in both models.

Interestingly, our MD simulations indicate a conformational change in Aβ_40_ upon binding to APP‐C31. As displayed in Figure 2g, the secondary structure of Aβ_40_ spanning from His13 to Asp23 becomes disordered, which is distinct from its monomeric form exhibiting an α‐helix in the same region (Figure 2f). In particular, the self‐recognition site of Aβ_40_ highlighted in purple adopts an extended conformation. The Ramachandran plots for the amino acid residues in the self‐recognition site (Leu17 to Ala21) present their dihedral angles mostly in the region of a β‐sheet and an extended helix,^[^ ^57^ ^]^ as summarized in Figure S18, Supporting Information. This observation suggests that the conformation of Aβ_40_ can be easily converted to a β‐strand in the presence of APP‐C31. Structural changes may occur during protein association, but the extent of change we observed is notable and offers additional explanations for the dominance of the entropy term in the binding free energy. There must be a lot of intra‐ and intermolecular displacements of hydrogen bond networks and van der Waals and electrostatic interactions upon complexation.^[^ ^3^ ^]^ Although we cannot explain what kinds of interactions positively and negatively contribute to the term of ∆*H*, a negative sign of ∆*H* obtained by the ITC analyses indicates the favorable non‐covalent interactions with ensemble structures. As a possible scenario with our ITC and MD results, we reasoned that the favorable entropic contribution could originate from both the liberation of water molecules that were hydrogen bonded to the polar sidechain such as Gln15,^[^ ^58^ ^]^ as well as the enhanced conformational degree of freedom (e.g., conformational entropy) of the extended backbone conformation. The transition to the extended motif increases the intrastrand conformational entropy which will help to compensate for the translational entropy penalty that must be paid during the hetero‐dimer formation. Ultimately, in addition to intermolecular ionic and hydrophobic interactions and hydrogen bonds, such structural alteration in Aβ can modify its oligomerization and fibrilization, as the self‐recognition site is critically involved in Aβ aggregation.^[^ ^7^, ^8^ ^]^ Overall, the results and observations by our MD simulations validate that APP‐C31 possibly interacts with the *N*‐terminal region and the self‐recognition site of Aβ_40_, which may induce its conformational transition.

### 2.4 Interactions of APP‐C31 with Metal Ions and Metal–Aβ_40_ and Its Impact on Metal–Aβ_40_ Aggregation

We assessed the interactions of APP‐C31 with metal ions and metal‐bound Aβ_40_ that are abnormally accumulated under AD‐affected conditions.^[^ ^7^, ^23^, ^24^, ^25^, ^26^, ^27^ ^]^ Metal ions such as Cu(I/II) and Zn(II) are reported to bind to the *N*‐terminal region of Aβ, thereby affecting its aggregation pathways.^[^ ^7^, ^23^, ^24^, ^25^, ^26^, ^27^, ^28^ ^]^ We questioned whether APP‐C31 can sequester the metal ion from metal–Aβ_40_ or form a ternary complex. The binding affinity of APP‐C31 to Cu(II) and Zn(II) was probed by ITC, as shown in Figure S19, Supporting Information. Upon titration of Cu(II) or Zn(II) to APP‐C31, two ITC peaks were indicated in the earlier thermograms. A sharp ITC peak was followed by a broad ITC peak, suggesting that metal binding to APP‐C31 caused its aggregation based on previously reported observations.^[^ ^59^ ^]^ Thus, although we could not determine the thermodynamic parameters of intermolecular interactions, the apparent values of the Gibbs free energy change (Δ*G*), the enthalpy change (Δ*H*), the entropy change (Δ*S*), and the dissociation constant (*K*_d_) were obtained. When Cu(II) was titrated to APP‐C31, an exergonic reaction was observed [Δ*G* = −6.5 (±0.1) kcal mol^−1^; Δ*H* = −0.5 (±0.1) kcal mol^−1^; *T*Δ*S* = 6.0 (±0.1) kcal mol^−1^] with the *K*_d_ value of 8.7 (±1.5) µm. Similarly, APP‐C31 binding to Zn(II) was also exergonic [Δ*G* = −6.2 (±0.1) kcal mol^−1^; Δ*H* = 0.3 (±0.1) kcal mol^−1^; *T*Δ*S* = 6.5 (±0.1) kcal mol^−1^]. The *K*_d_ value for Zn(II)–APP‐C31 was measured to be 18.0 (±4.6) µm. The increase in entropy observed here is likely due to the release of water molecules that were tightly bound to the metal ion in the first solvent shell and become free water molecules as the metal ion binds to the peptide.^[^ ^60^ ^]^ Given that the *K*_d_ values of Cu(II)–Aβ and Zn(II)–Aβ are 10^−10^ and 10^−6^ m, respectively,^[^ ^7^, ^23^, ^26^, ^27^, ^61^, ^62^ ^]^ APP‐C31 may not be able to extract the metal ion from metal‐bound Aβ.

**Figure 3:**
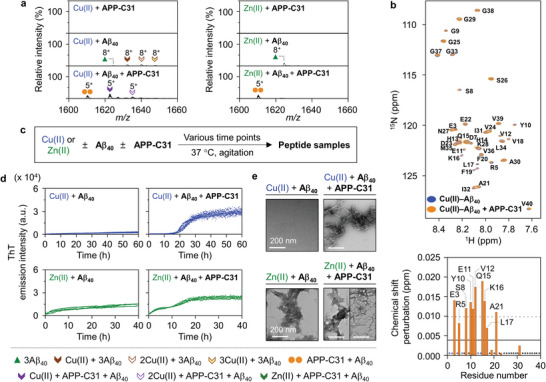
Interactions of APP‐C31 with metal‐treated Aβ_40_ and its influence on metal‐induced Aβ_40_ aggregation. a) Interactions of APP‐C31 with metal‐treated Aβ_40_ observed by ESI–MS. The full MS spectra are depicted in Figures S21 and S22, Supporting Information. Conditions: [APP‐C31] = 100 µm; [Aβ_40_] = 100 µm; [CuCl_2_ or ZnCl_2_] = 100 µm; 20 mm ammonium acetate, pH 7.3; 37 °C; 2 h incubation; no agitation. The samples were diluted by tenfold prior to injection to the mass spectrometer. b) Interaction of APP‐C31 with Cu(II)‐treated Aβ_40_ analyzed by 2D ^1^H–^15^N HSQC NMR spectroscopy (700 MHz). The average of CSPs and the average + one standard deviation are indicated with solid and dashed lines, respectively. Conditions: [^15^N‐labeled Aβ_40_] = 40 µm; [APP‐C31] = 200 µm; [CuCl_2_] = 20 µm; 20 mm HEPES, pH 7.4; 10 °C. The amino acid residues indicated in blue or black asterisks represent the residues that were unresolved or not significantly shifted, respectively. c) Scheme of the aggregation experiments. d) Degree on the aggregation of metal‐added Aβ_40_ with or without APP‐C31 detected by the ThT assay. Experiments were conducted in triplicate. e) Morphology of the peptide aggregates generated by incubation of Cu(II) or Zn(II)‐treated Aβ_40_ with and without APP‐C31 for 60 or 40 h, respectively, monitored by TEM. Scale bar = 200 nm. Conditions: [APP‐C31] = 20 µm; [Aβ_40_] = 20 µm; [CuCl_2_] = 18 µm; [ZnCl_2_] = 20 µm; 20 mm HEPES, pH 7.4, 150 mm NaCl; 37 °C; constant agitation (559 cpm).

When Cu(II) or Zn(II) binding to APP‐C31 was monitored by ESI–MS, the low peak corresponding to [Cu(II) + APP‐C31]^4+^ was observed, indicative of the Cu(II)–APP‐C31 complexation, whereas Zn(II)‐bound APP‐C31 was not observed under our experimental conditions (Figure S20, Supporting Information). It is likely due to the disruption of metal–APP‐C31 interactions during the transition from solution to the gas phase used for ESI–MS. Different from peptide–peptide interactions, only four to six non‐covalent bonds regulate metal–peptide complexation and, thus, the bonds can be relatively easily broken in electrospray droplets.^[^ ^63^ ^]^ Despite the experimental limitation to observe metal–peptide complexes, the interaction of APP‐C31 with Cu(II)‐treated Aβ_40_ was verified by ESI–MS. As illustrated in **Figure** **3**a and Figure S21, Supporting Information, the sample containing APP‐C31, Cu(II), and Aβ_40_ in a molar ratio of 1:1:1 presented the peaks assigned to [Cu(II) + Aβ_40_ + APP‐C31]^5+^ and [2Cu(II) + Aβ_40_ + APP‐C31]^5+^. The peak corresponding to [Zn(II) + Aβ_40_ + APP‐C31]^5+^ with a very low intensity was also detected when APP‐C31 was added to Zn(II)–Aβ_40_ (Figure S22, Supporting Information). Moreover, based on our observation on the formation of the ternary complexes by ESI–MS, the interaction between Cu(II)–Aβ_40_ and APP‐C31 was further probed by 2D ^1^H–^15^N HSQC NMR spectroscopy. As described in Figure S23, Supporting Information, when Cu(II) was treated to a solution of uniformly ^15^N‐labeled Aβ_40_, noticeable changes in CSPs were identified at several regions of Aβ_40_ (e.g., Asp7, Ser8, Tyr10, Glu11, Gln15, Lys16, Val18, Phe19, Gly29, Ala30, and Ile31). In addition, the treatment of Cu(II) induced an overall reduction in the signal intensity of ^15^N‐labeled Aβ_40_, and further decreased intensities at the *N*‐terminal half part of Aβ_40_ due to its paramagnetic effects. These results indicate intermolecular interactions between Cu(II) and Aβ_40_ as well as complexation, consistent with previous studies.^[^ ^64^, ^65^ ^]^ When APP‐C31 was added to Cu(II)–Aβ_40_, significant changes in CSPs were observed at the Glu3, Arg5, Ser8, Tyr10, Glu11, Val12, Gln15, Lys16, Leu17, and Ala21 residues in ^15^N‐labeled Aβ_40_ (Figure 3b). All these results suggest APP‐C31 binding to the *N*‐terminal and the central self‐recognition sites of Cu(II)–Aβ_40_. Therefore, our studies manifest that APP‐C31 can directly interact with metal ions and form a ternary complex with metal–Aβ_40_.

Moving forward, the influence of APP‐C31 on the aggregation of metal–Aβ_40_ was investigated by the ThT assay and TEM. When Cu(II) was incubated with Aβ_40_, aggregates were not significantly generated up to 60 h, as depicted in Figure 3c,d. In the presence of APP‐C31, the rapid amyloid aggregation of Cu(II)–Aβ_40_ was induced displaying the *t*_lag_ and *t*_1/2_ values of 18.4 (±0.8) h and 23.2 (±1.1) h, respectively. Linear amyloid fibrils with short lengths were only detected from the sample of Cu(II)–Aβ_40_ with APP‐C31, as presented in Figure 3e. It is noteworthy that the noticeable fibrillary species were not obtained from Cu(II)–Aβ_40_, contrary to our earlier work,^[^ ^66^ ^]^ which may result from the difference in manufacturers, buffer and salt concentrations, consumables, and instruments for Aβ aggregation.^[^ ^8^, ^40^, ^66^, ^67^ ^]^ In the case of Zn(II)–Aβ_40_, the aggregation was initiated without the lag phase, which is a typical nucleation‐independent amorphous aggregation, while APP‐C31 altered Zn(II)–Aβ_40_ aggregation pathways exhibiting the biphasic growth of the aggregation (Figure 3d). TEM results indicated that the addition of APP‐C31 produced a mixture of amorphous aggregates and amyloid fibrils, different from amorphous aggregates mainly obtained by incubation of Zn(II)– Aβ_40_ (Figure 3e). It should be emphasized that APP‐C31 with Cu(II) and Zn(II) in the absence of Aβ_40_ did not change ThT fluorescence and, thus, amyloid fibrils did not form with metal‐treated APP‐C31 under our experimental conditions (Figure S24, Supporting Information). Only small globular aggregates were monitored from Cu(II)‐added APP‐C31 by TEM (Figure S25, Supporting Information). Overall, our investigations reveal that APP‐C31 modulates the aggregation of Cu(II)–Aβ_40_ and Zn(II)–Aβ_40_ in a distinct manner.

### 2.5 Cytotoxicity of Aβ_40_ Associated with APP‐C31

**Figure 4:**
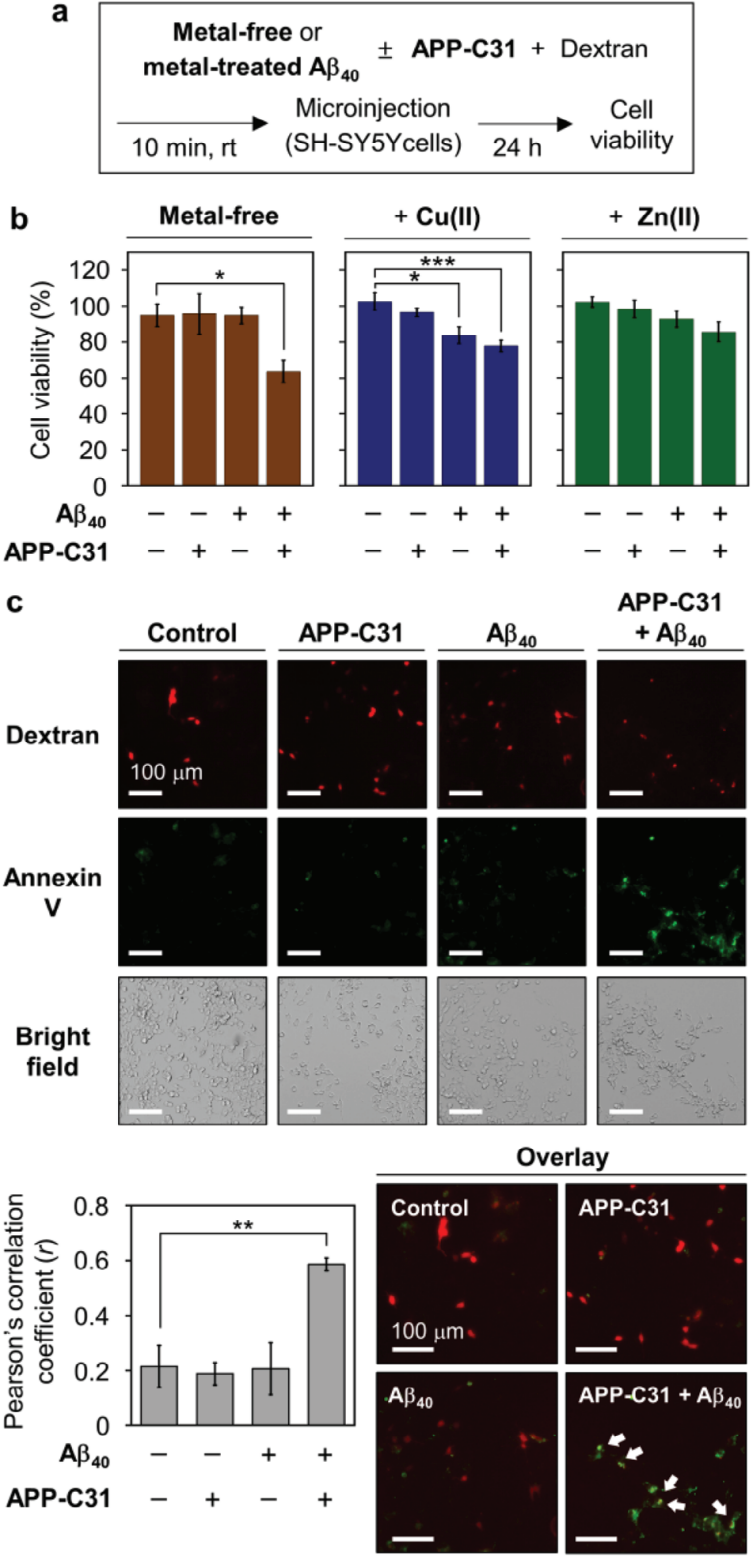
Influence of APP‐C31 on the toxicity of intracellular Aβ_40_. a) Scheme of the microinjection experiments. b) Cell viability of SH‐SY5Y cells microinjected with either APP‐C31, Aβ_40_, or both in the absence and presence of metal ions. Cell survival measurements were described in detail in the experimental section. Conditions: [Aβ_40_] = 10 µm; [APP‐C31] = 10 µm; [CuCl_2_ or ZnCl_2_] = 10 µm; [Texas Red‐labeled dextran] = 0.4 mg mL^−1^; 24 h incubation. All values denote mean ± s.e.m. *p < 0.05; ***p < 0.001; *n* = 3–5; Student's *t*‐test. c) Apoptosis monitored using Alexa Fluor 488‐tagged annexin V. Pearson's correlation coefficients were calculated to present the colocalization of the fluorophore‐tagged dextran and Annexin V (mean ± s.e.m, **p < 0.01; *n* = 3–5; Student's *t*‐test). White arrows indicate apoptotic cells microinjected with peptide samples. Conditions: [Aβ_40_] = 10 µm; [APP‐C31] = 10 µm; [Texas Red‐labeled dextran] = 0.4 mg mL^−1^; 24 h incubation. Scale bar = 100 µm.

As APP‐C31 accelerates the aggregation of intracellular Aβ_40_ and triggers the deposition of Aβ_40_ aggregates especially at the nuclear and perinuclear regions, as illustrated in Figure 1, its influence on the cytotoxicity associated with intracellular Aβ_40_ was monitored by microinjection experiments. Aβ_40_ species with and without APP‐C31 and metal ions were injected into living cells, as described in **Figure** **4**a, with Texas Red‐labeled dextran which is an indicator for the cells treated with the samples.^[^ ^34^ ^]^ After 24 h incubation, cell viability was determined by counting the Texas Red‐positive cells following previously reported methods.^[^ ^68^ ^]^ After injection with either APP‐C31 or Aβ_40_, about 95% of the cells survived under our experimental conditions, as displayed in Figure 4b. When both APP‐C31 and Aβ_40_ were administered inside the cells, cell death was enhanced by ≈40%. In the case of Cu(II)‐ and Zn(II)‐added Aβ_40_, the viability was 84% and 93%, respectively. Upon addition of metal‐treated Aβ_40_ with APP‐C31 inside the cells, the presence of Cu(II)–Aβ_40_ or Zn(II)–Aβ_40_ with APP‐C31 induced relatively more cytotoxicity exhibiting 78% and 86% viability, respectively. Thus, our microinjection studies demonstrate that APP‐C31 aggravates the cytotoxicity of intracellular Aβ_40_ with and without metal ions to different degrees.

Thus far, the role of APP‐C31 in inducing apoptosis and increasing the presence of Aβ has been suggested;^[^ ^12^, ^13^, ^14^ ^]^ however, it remains elusive. We investigated whether the treatment of both APP‐C31 and Aβ_40_ inside the cells induces apoptosis by applying live‐cell imaging experiments using annexin V staining. Annexin V conjugated with Alexa Fluor 488 can stain externalized phosphatidylserine in apoptotic cells, thereby emitting green fluorescence.^[^ ^69^ ^]^ The overlap between green and red fluorescence from annexin V and Texas Red‐labeled dextran, respectively, indicates the cells that are injected with the samples and undergo apoptosis. The degree of the colocalization between green and red fluorescence was analyzed by Pearson's coefficient (*r*), a type of correlation coefficient that represents the strength of the association between the two variables.^[^ ^70^ ^]^ When APP‐C31 or Aβ_40_ was added inside the cells followed by 24 h incubation, the cells did not significantly show the green fluorescence of annexin V, as displayed in Figure 4c. The *r* value was ≈0.2, indicative of the poor association between apoptosis and the internalization of APP‐C31 or Aβ_40_ under our experimental conditions. Notably, the injection of both APP‐C31 and Aβ_40_ into the cells resulted in the intense green fluorescence of annexin V with an *r* value of ≈0.6. Clearly, these results support that the intracellular presence of the two peptides can cause apoptosis.

**Figure 5:**
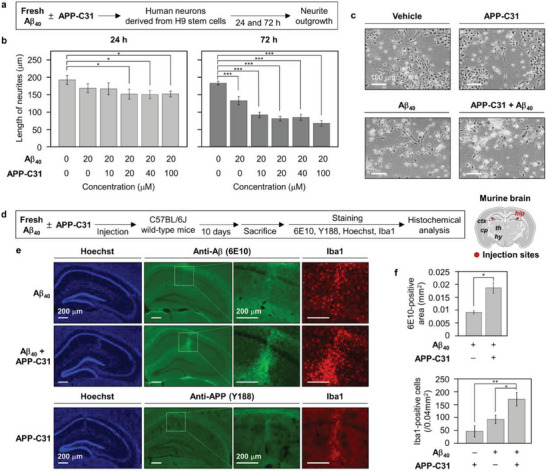
Effects of APP‐C31 on the neurodegeneration and inflammatory response induced by Aβ_40_. a) Scheme of the experiments with human neuronal stem cells. b) Analysis of the average length of neurites as a function of APP‐C31's concentration. c) Representative images of neurons after 72 h incubation with either 20 µm of APP‐C31, Aβ_40_, or both. 15 neurons were analyzed per each condition. The average length of neurites in the presence of various concentrations of APP‐C31 only is reported in Figure S26, Supporting Information. Conditions: [Aβ_40_] = 20 µm; [APP‐C31] = 0, 10, 20, 40, and 100 µm; incubation for 24 and 72 h. Scale bar = 100 µm. Data are represented as mean ± s.e.m. *p < 0.05; ***p < 0.001; Student's *t*‐test. d) Scheme of in vivo experiments and injection sites in the brain. Hippocampus (*hip*), cortex (*ctx*), thalamus (*th*), caudate putamen (*cp*), and hypothalamus (*hy*) are shown. e) Microscopic images of the hippocampi of C57BL/6J mice injected with Aβ_40_, APP‐C31, or both, visualized by immunohistochemical analyses [primary antibodies, 6E10 (anti‐Aβ antibody; green), Y188 (anti‐APP antibody; green), Iba1 (anti‐microglial antibody; red)] or fluorescent dye staining (Hoechst for nucleus). Scale bar = 200 µm. f) Quantification results of the 6E10‐positive area and Iba1‐positive cells, calculated based on the fluorescence signals. All values denote mean ± s.e.m. *p < 0.05, **p < 0.01; *n* = 3 (for 6E10 staining), *n* = 5 (for Y188 staining), and *n* = 5 (for Iba1 staining); Student's *t*‐test.

Next, the effect of Aβ_40_ species on human neuronal growth was investigated in the presence of various concentrations of APP‐C31 (**Figure** **5**a–c and Figure S26, Supporting Information). Human neurons differentiated from H9 human embryonic stem cells were prepared, and peptides were introduced into the media of human neurons, as described in Figure 5a. After 24 h and 72 h incubation, the length of neurites was measured to monitor neuronal growth. Neurons incubated with Aβ_40_ for 24 h exhibited slightly shortened neurites by ≈10%, compared to vehicle‐added neurons (Figure 5b). The averaged neurite length further decreased when Aβ_40_ with stoichiometric or suprastoichiometric amounts of APP‐C31 was treated to neurons. After 72 h incubation, neurons incubated with Aβ_40_ displayed the reduced length of neurites by about 35%, compared to the control. More noticeably, the addition of Aβ_40_ and various concentrations of APP‐C31 to neurons led to the reduction of the neurite length by 50% to 65% and exhibited the fragmentation of neurites. These observations suggest that the Aβ_40_‐induced degeneration of neurites can be intensified by APP‐C31.

On the basis of these results, the impact of APP‐C31 on the toxicity of Aβ_40_ species was further probed in vivo (murine brains) through immunohistochemical investigations. As depicted in Figure 5d, Aβ_40_ was directly injected with or without APP‐C31 into the hippocampus, where protein deposits and *C*‐terminally cleaved APP are dominantly observed in the AD‐affected brain.^[^ ^3^, ^6^ ^]^ After 10 days, the brain sections were stained with various fluorescent dyes or antibodies detecting the nucleus, Aβ, APP‐C31, and microglia. Compared to the hippocampus of the mice injected with Aβ_40_, the 6E10‐positive area was significantly enhanced when both Aβ_40_ and APP‐C31 were injected (Figure 5e,f). These results imply the potential involvement of APP‐C31 in the assembly of Aβ_40_ in the brain. It should be noted that the fluorescent signal of thioflavin‐S in response to amyloid fibrils was not significantly detected (data not shown), which may be due to the slow fibrilization of Aβ_40_ under our experimental settings.^[^ ^3^ ^]^ Moreover, it was found that the number of the Iba1‐positive microglial cells^[^ ^5^ ^]^ showed a 3.7‐fold increase upon injection of both Aβ_40_ and APP‐C31, compared to that of Aβ_40_ only. Given the association of the enhanced microglial recruitment with the activation of immune responses,^[^ ^3^, ^4^ ^]^ these results manifest that the Aβ_40_‐induced inflammatory response can be further amplified by addition of APP‐C31. Taken together, our in vitro and in vivo studies manifest that APP‐C31 can detrimentally impact the toxicity of Aβ_40_.

## 3 Conclusion

APP‐C31 is generated by cleaving the intracellular *C*‐terminal region of APP by caspases.^[^ ^1^ ^]^ This peptide is suggested to be a toxic APP fragment. The role of APP‐C31 in synaptic damage and neurodegeneration has been identified.^[^ ^1^, ^10^, ^12^, ^13^ ^]^ An inter‐relationship among the activation of caspases, the intracellular *C*‐terminal cleavage of APP, and the Aβ‐induced toxicity toward amyloid pathology has been recently proposed.^[^ ^5^, ^11^, ^12^, ^13^ ^]^ Moreover, it is likely that APP‐C31 may alter the cellular Aβ_42_‐to‐Aβ_40_ ratio that could affect amyloid aggregation and neurotoxicity.^[^ ^14^, ^71^ ^]^ But to date, it is not known whether APP‐C31 directly interacts with Aβ and alters its misfolding and aggregation that ultimately leads to the Aβ‐related toxicity. We report for the first time that APP‐C31 directly affects the aggregation and toxicity of Aβ. We found that metal ions such as Cu(II) and Zn(II) that are known to bind to Aβ in AD‐affiliated brains have notable influence without changing the overall impact decisively.

Our work illustrates that APP‐C31 can form a hetero‐dimer complex with Aβ_40_ through the contacts onto the *N*‐terminal region and the self‐recognition site of Aβ_40_. Our computer simulations suggest that upon complexation with APP‐C31, Aβ_40_ unfolds the α‐helical segment at the self‐recognition site. This structural rearrangement facilitates the β‐sheet formation that is critical for the aggregation of Aβ.^[^ ^7^, ^8^, ^72^ ^]^ Notably, APP‐C31 promotes the aggregation of Aβ_40_ possibly via affecting the primary nucleation step. The aggregation kinetics of Aβ_42_ is slightly influenced by APP‐C31, but a noticeable change in the morphology of the resultant Aβ_42_ aggregates is observed. These results imply that APP‐C31 may interact with Aβ_42_ and cause its polymorphic amyloidogenesis. We speculate that the faster fibrilization kinetics and higher aggregation propensities of Aβ_42_ than Aβ_40_ may lead to fewer intermolecular interactions between APP‐C31 and Aβ_42_.^[^ ^73^ ^]^ In the near future, we will conduct high‐resolution structural studies of Aβ_42_ aggregates with APP‐C31 which can advance our better understanding of the role of APP‐C31 in the aggregation of Aβ peptides.

In the presence of metal ions, APP‐C31 accelerates the aggregation of Cu(II)–Aβ_40_ and modifies the aggregation pathways of Zn(II)–Aβ_40_. In living cells, larger Aβ_40_ aggregates are intracellularly produced in the presence of APP‐C31. APP‐C31 can induce the perinuclear and intranuclear deposition of Aβ_40_ aggregates. APP‐C31 may assist in translocating Aβ_40_ into the perinuclear region or nuclei, consistent with previous observations that the *C*‐terminal fragments of APP can transfer into nuclei after being complexed with other proteins.^[^ ^74^, ^75^ ^]^ This is further supported by the recent findings that perinuclear Aβ aggregates are colocalized with the *C*‐terminal region of APP.^[^ ^15^, ^16^ ^]^ Moreover, Aβ_40_ incubated with APP‐C31 significantly suppresses the outgrowth of neurites in human neurons and notably amplifies the immune response in vivo (murine brains). Clearly, APP‐C31 can exacerbate the toxicity mediated by Aβ_40_. The toxicity of metal‐bound Aβ_40_ is less than that of metal‐free Aβ_40_ in the presence of APP‐C31, suggesting that the altered structure and aggregation pathways of Aβ by metal ions may affect their interactions with APP‐C31 and, concomitantly, induce different toxicity pathways. Overall, our combined experimental and theoretical studies demonstrate that the interactions of APP‐C31 with both metal‐free Aβ_40_ and metal–Aβ_40_ transform their aggregation and toxicity profiles.

## 4 Experimental Section

### Materials and Methods

All reagents were purchased from commercial suppliers and used as received unless otherwise stated. Aβ_40_ (DAEFRHDSGYEVHHQKLVFFAEDVGSNKGAIIGLMVGGVV) and Aβ_42_ (DAEFRHDSGYEVHHQKLVFFAEDVGSNKGAIIGLMVGGVVIA) were obtained from Peptide Institute, Inc. (Osaka, Japan) that was purified by high‐performance liquid chromatography (HPLC) using YMC Pack ODS‐A (YMC CO., LTD., Kyoto, Japan) and Agilent ZORBAX 300SB‐C18 columns (Agilent, Santa Clara, CA, USA), respectively, or Anaspec (Fremont, CA, USA). APP‐C31 (AAVTPEERHLSKMQQNGYENPTYKFFEQMQN) and ^HF647^Aβ_40_ were purchased from AnaSpec. Double‐distilled water (ddH_2_O) used for all experiments was obtained from a Milli‐Q Direct 16 system (Merck KGaA, Darmstadt, Germany). Trace metal contamination was removed from the solutions used for the aggregation experiments by treating Chelex (Sigma‐Aldrich, St. Louis, MO, USA) overnight. The concentrations of peptides were determined by an Agilent 8453 UV−Visible spectrophotometer (Agilent). Experiments by ESI–MS were performed by an Agilent 6530 Accurate Mass Quadrupole Time‐of‐Flight (Q‐TOF) mass spectrometer with an ESI source (Agilent). 2D ^1^H‐^15^N HSQC NMR spectroscopy was conducted by an AVANCE II‐900 MHz or 700 MHz NMR spectrometer [Bruker BioSpin, Rheinstetten, Germany; Korea Basic Science Institute (KBSI), Ochang, Republic of Korea] equipped with a cryogenic probe. NMR studies of APP‐C31 with Aβ_40_ fibrils were carried out on a Bruker Avance II 800 MHz NMR spectrometer (Bruker BioSpin; KBSI) equipped with a cryogenic probe. ITC was performed by a VP‐ITC instrument equipped with a motor‐driven syringe (Malvern Panalytical, Malvern, UK). The secondary structure of APP‐C31 was analyzed by a JASCO‐815 150‐L CD spectropolarimeter [Jasco Inc., Tokyo, Japan; KAIST Analysis Center for Research Advancement (KARA), Daejeon, Republic of Korea]. Morphological changes of peptide aggregates were monitored by a Tecnai F20 transmission electron microscope (FEI Company, Eindhoven, Netherlands; KARA). A Synergy Neo2 Hybrid Multi‐Mode microplate reader (Biotek, Winooski, VT, USA) was employed to measure the fluorescence for the ThT assay. Live‐cell microinjection experiments were performed by the combined system composed of an InjectMan 4 micromanipulator (Eppendorf, Hamburg, Germany) and a Femtojet 4i microinjector (Eppendorf). Fluorescence analysis of living cells was conducted by an EVOS FL fluorescence microscope (Advanced Microscopy Group, Bothell, WA, USA). Confocal microscopic images were taken by Zeiss LSM 880 (Zeiss, Zena, Germany; KARA).

### Preparation of APP‐C31 and Aβ

APP‐C31 and Aβ were dissolved in ddH_2_O and ammonium hydroxide (NH_4_OH; 1% w/w, aq), respectively. After lyophilizing the resulting solutions, the peptides were stored at −80 °C. A stock solution of Aβ was prepared by dissolving the lyophilized peptide with NH_4_OH (1% w/w, aq; 10 µL) and diluting with ddH_2_O, as previously reported.^[^ ^76^ ^]^ A stock solution of APP‐C31 was prepared by dissolving the lyophilized peptide in ddH_2_O. The concentration of Aβ was determined by measuring the absorbance of the solution at 280 nm (*ε*_280_ = 1,450 M^−1^cm^−1^ for Aβ_40_; *ε*_280_ = 1,490 M^−1^cm^−1^ for Aβ_42_).^[^ ^76^ ^]^ As the extinction coefficient of APP‐C31 has not been reported, the concentration of APP‐C31 was determined based on the theoretical extinction coefficient (*ε*_280_ = 2,980 M^–1^cm^–1^) obtained from the ExPASy ProtParam online tool.^[^ ^77^ ^]^

### ThT Fluorescence Assay

Aβ (20 µm), APP‐C31 (20, 100, and 200 µm), Aβ_40_ seeds (5% v/v), and metal ions (18 µm for CuCl_2_; 20 µm for ZnCl_2_) were incubated with ThT (5 µm) at 37 °C with constant agitation (559 cpm) in 20 mm HEPES [4‐(2‐hydroxyethyl)−1‐piperazineethanesulfonic acid], pH 7.4, 150 mm NaCl. Aβ_40_ seeds were prepared by incubation of Aβ_40_ (20 µm) for 40 h at 37 °C with constant agitation (250 rpm) in 20 mm HEPES, pH 7.4, 150 mm NaCl followed by ultrasonication with a Q55 sonicator (Qsonica, Newtown, CT, USA). All experiments were prepared in half‐area 96‐well plates that have a non‐binding surface (Corning, Kennebunk, ME, USA). The fluorescence intensity was measured by a microplate reader (*λ*_ex_ = 445 nm; *λ*_em_ = 485 nm). The kinetic parameters of Aβ aggregation were acquired by fitting the emission curves using a modified Boltzmann‐sigmoidal Equation (1) in the Origin software:

(1) $$F\;\left(t\right)=F_{0}\;+\frac{A}{1\;+\;\;e^{-k\left(t-t_{1/2}\right)}}$$

(2) $$\;t_{lag}=\;t_{1/2}-2\left(1/k\right)$$

*F*_0_ and A are the initial fluorescence intensity and amplitude, respectively.^[^ ^40^ ^]^ *t*_1/2_ indicates the half time when fluorescence reaches 50% of its maximum intensity.^[^ ^40^ ^]^ *k* is the rate constant of the elongation phase. The lag time, *t*_lag_, was defined using an Equation (2).^[^ ^40^ ^]^ Experiments were performed in triplicate. Data are presented as mean ± s.e.m. (standard error of the mean) of all experiments.

### Gel/Western Blot

Samples containing Aβ (25 µm) with and without APP‐C31 (25 µm) in the absence and presence of CuCl_2_ or ZnCl_2_ (25 µm) were incubated in 20 mm HEPES, pH 7.4, 150 mm NaCl at 37 °C with constant agitation. After 24 h incubation, The samples (10 µL) were separated on a 10%–20% Tris‐tricine gel (Invitrogen, Grand Island, NY, USA). Following separation, the proteins were transferred onto nitrocellulose membranes and blocked with bovine serum albumin (BSA, 3% w/v, Biosesang, Seongnam, Republic of Korea) in Tris‐buffered saline (TBS) containing 0.1% v/v Tween‐20 (TBS‐T) for 3 h at room temperature. Then, the membrane was incubated with a primary antibody [an anti‐Aβ antibody (6E10; 1:2,000, Covance, Princeton, NJ, USA) or an anti‐APP *C*‐terminus antibody (Y188; 1:2,000, Abcam, Cambridge, MA, USA)] for 4 h at room temperature. After washing with TBS‐T three times (7 min each), the horseradish peroxidase‐conjugated goat anti‐mouse secondary antibody (1:2,500 for 6E10; Cayman, Ann Arbor, MI, USA) or the goat anti‐rabbit secondary antibody (1:2,000 for Y188 antibody; Promega, Madison, WI, USA) in the solution of BSA (2% w/v in TBS‐T) was added to the membrane and incubated for 1 h at room temperature. The membrane was visualized by an imaging system with a homemade ECL kit.^[^ ^78^ ^]^ To monitor both peptides in the samples, the same membrane was stripped after the visualization by treating it with hydrogen peroxide (H_2_O_2_) for 30 min at room temperature followed by washing four times with TBS‐T for 10 min each, blocking with the solution of BSA [3% w/v in TBS‐T (0.01% v/v)], and incubating with the other antibody.

### TEM

Samples for TEM measurements were prepared based on previously published methods.^[^ ^76^ ^]^ Glow‐discharged grids (Formvar/Carbon 300‐mesh, Electron Microscopy Sciences, Hatfield, PA, USA) were treated with the resultant samples for 2 min at room temperature. The excess sample was removed using a filter paper. Each grid was washed three times with ddH_2_O and incubated with uranyl acetate (1% w/v ddH_2_O; 5 µL) for 1 min. After removing excess uranyl acetate, the grids were dried overnight at room temperature. Images of each grid were taken at 200 kV with a magnification of 29,000×. For the TEM analysis, the location of samples on the grids was randomly selected for taking more than 15 images per each grid.

### CD Spectroscopy

APP‐C31 (0.4 mg mL^−1^) was incubated for 0 and 40 h at 37 °C in 20 mm HEPES, pH 7.4, 150 mm NaF. The samples were prepared in a half‐area 96‐well plate that has a non‐binding surface (Corning). The CD spectra of APP‐C31 samples were collected in the range from 200 to 250 nm with a cell path length of 0.5 mm. The digital integration time, the bandwidth, and the scanning speed were 4 s, 2 nm, and 20 nm min^−1^, respectively. Each spectrum was smoothed by Fourier transforms.

### Cell Culture and Microinjection

The human neuroblastoma SH‐SY5Y cell line was purchased from the American Type Culture Collection (ATCC; Manassas, VA, USA). The cell line was maintained in media containing 50% v/v Dulbecco's Modified Eagle Medium and 50% v/v Nutrient Mixture F12 (phenol red‐free DMEM/F12; GIBCO, Grand Island, NY, USA) supplemented with 10% v/v fetal bovine serum (GIBCO) and 100 U mL^−1^ penicillin‐streptomycin (GIBCO). Cells were grown and maintained at 37 °C in a humidified atmosphere with 5% CO_2_. The cells used for the studies did not indicate mycoplasma contamination. Cells were plated on an imaging dish (35 mm; 1.0 × 10^5^ cells per 1 mL) and incubated for 48 h before microinjection. The solutions of peptides were centrifuged at 5,000 rpm for 1 min to remove large aggregates that can clog a microinjection glass capillary. The solutions of either Aβ_40_ (10 µm), APP‐C31 (10 µm), or both peptides in the absence and presence of CuCl_2_ or ZnCl_2_ (10 µm) were mixed with dextran labeled with Texas Red (0.4 mg mL^−1^, 3000 MW; Invitrogen, Carlsbad, CA, USA) followed by injection into the cells using an InjectMan 4 micromanipulator and a Femtojet 4i microinjector. The injection pressure (*P*_i_), the compensation pressure (*P*_c_), and the injection time (*t*_i_) were set to 50 hPa, 20 hPa, and 0.2 s, respectively. Cell viability was determined based on the previously reported procedures.^[^ ^68^ ^]^ After microinjection, the number of Texas Red‐positive cells was first counted after 3 h and it was assigned as 100% of cell viability to exclude the cells damaged by microinjection. Cells were counted after 24 h incubation and cell viability (%) was calculated. At least 100 cells from each sample were injected and analyzed. Experiments were performed at least in triplicate. Data are represented as mean ± s.e.m (standard error of the mean) of three independent experiments. Fluorescence analysis of the cells was conducted by a fluorescence microscope with an RFP light cube [excitation at 531 (±40) nm and emission at 593 (±40) nm].

### Annexin V Staining

SH‐SY5Y cells were plated on an imaging dish (35 mm; 0.3 × 10^5^ cells per 1 mL) and incubated for 48 h. The solutions of either Aβ_40_ (10 µm), APP‐C31 (10 µm), or both peptides were mixed with dextran labeled with Texas Red, and the resultant solutions were microinjected into the cells, as described above. After 24 h incubation, the cells were washed with annexin‐binding buffer and stained with annexin V‐Alexa Fluor 488 for detecting apoptosis following the manufacturer's instruction (FITC Annexin V/Dead Cell Apoptosis Kit; V13241, Invitrogen). Fluorescent responses of annexin V and Texas Red‐labeled dextran were identified by a microscope with a GFP light cube [excitation at 470 (±22) nm and emission at 525 (±50) nm] and a Cy5 light cube [excitation at 628 (±40) nm and emission at 685 (±40) nm], respectively. At least five randomly selected fields per conditions were captured with a microscope. Pearson's correlation coefficient for the indication of co‐localization was calculated with the Coloc2 plugin in Fiji software.^[^ ^79^ ^]^

### Confocal Microscopy

All experiments were conducted with Aβ_40_ conjugated to the HiLyte Fluor 647 (HF647) fluorophore at the *N*‐terminus (Anaspec). The concentration of ^HF647^Aβ_40_ was determined by measuring the absorbance at 649 nm (*ε*_649_ = 250,000 M^–1^cm^–1^).^[^ ^36^ ^] HF647^Aβ_40_ (10 µm) with or without APP‐C31 (10 µm) was microinjected into SH‐SY5Y cells followed by incubation for 24 h. ^HF647^Aβ_40_ aggregates were monitored from three or more randomly selected fields per condition with a lateral resolution of 120 nm. A series of the Z‐stack images was acquired with the step size of 2 µm. The size of ^HF647^Aβ_40_ aggregates was determined by the “analyze particles” program of Fiji software based on the previously reported reports.^[^ ^37^ ^]^ All pixels above the threshold value were selected to calculate the area of ^HF647^Aβ_40_ aggregates. This analysis included ^HF647^Aβ_40_ aggregates with the size larger than 0.05 µm^2^. To stain the nuclei of cells, cells were incubated with a SYTO16 green fluorescent nucleic acid stain dye (2.5 µm; Invitrogen) for 10 min. Images were obtained by adopting a confocal microscope (*λ*_ex_ = 488 nm for a nucleic acid dye; λ_ex_ = 633 nm for ^HF647^Aβ_40_).

### Neuronal Differentiation from Embryonic Stem Cells

Human neurons were differentiated from neural progenitor cells (NPCs), which are derived from human embryonic stem cells (hESCs), WA09 (also known as H9). hESCs were maintained on inactivated mouse embryonic fibroblasts (MEFs) in hESC‐media comprising DMEM/F12 (GIBCO) with MEM non‐essential amino acids (MEM NEAA; GIBCO), sodium bicarbonate (14 mm; Sigma‐Aldrich), L‐glutamine (1 mm; Sigma‐Aldrich), β‐mercaptoethanol (100 µm; Merck Millipore, Billerica, MA, USA), knockout serum replacement (20% v/v; GIBCO), and bFGF (10 ng mL^−1^; R&D systems, McKinley Place, MN, USA). MEFs were collected from E13.5 CrljOri:CD1 (ICR) mice (Orientbio, Seongnam, Republic of Korea) and inactivated with 10 µg mL^−1^ of mitomycin C (AG Scientific, San Diego, CA, USA) in 2 h. hESC colonies were dissociated with Collagenase IV and plated onto Petri dishes in hESC‐media to form embryoid bodies (EBs). At the following day, floating EBs were treated with LDN (Selleckchem, Huston, TX, USA) and SB‐431542 (Cayman) in DMEM/F12 + glutamax (GIBCO) with N2 and B27 supplements (GIBCO). The treatment was continued for a week, followed by plating onto growth factor reduced matrigel (BD biosciences)‐coated dishes in DMEM/F12 + glutamax (GIBCO) with N2 and B27 supplements (N2/B27 media) and laminin (1 mg mL^−1^; GIBCO). Within a few days, neural rosettes were manually picked and dissociated with Accutase (Innovative Cell Technologies, San Diego, CA, USA) and plated onto poly‐L‐ornithine/laminin‐coated dishes with NPC media (N2/B27 media with 20 ng mL^−1^ of bFGF). To differentiate NPCs into neurons, NPCs were plated onto poly‐L‐ornithine/laminin‐coated dishes in N2/B27 media in the presence of ascorbic acid (200 nm; Sigma‐Aldrich), dcAMP (500 mg mL^−1^; Selleckchem), BDNF (20 ng mL^−1^; Peprotech, London, UK), GDNF (20 ng mL^−1^; Peprotech), and laminin (1 mg mL^−1^), as previously described.^[^ ^80^ ^]^ Protocols describing the use of MEFs and hESCs were approved by the ethical requirements and regulations of the Institutional Review Board of KAIST (IRB #KA2020‐37 for MEFs and IRB #KA2018‐61 for hESCs).

### Measurement of Neurite Outgrowth

Human NPCs were differentiated into neurons for 2 d and the media was replaced by fresh media. Neurons were treated with either Aβ (20 µm), APP‐C31 (10, 20, 40, and 100 µm), or both peptides followed by incubation for 24 and 72 h. Under each condition, at least 5 randomly selected fields were captured with an Olympus IX71 microscope. The neurite of a neuron measured from the soma to the tip was analyzed using Neuron J program of Fiji software.^[^ ^81^ ^]^

### ESI–MS

APP‐C31 (100 µm) was incubated with Aβ_40_ (100 µm) in the absence and presence of CuCl_2_ or ZnCl_2_ (100 µm) for 2 h at 37 °C in 20 mm ammonium acetate, pH 7.3. The samples were prepared in microtubes (Eppendorf). Before injection into the mass spectrometer, the resultant samples were diluted by tenfold with LC‐grade H_2_O. The capillary voltage, the drying gas flow, and the gas temperature were set to 5.8 kV, 12 L min^−1^, and 300 °C, respectively. The measurements were conducted in triplicate.

### Stereotaxic Peptide Delivery in the Mouse Brain

All experiments were performed in accordance with approved animal protocols and guidelines established by Korea Research Institute of Bioscience and Biotechnology (KRIBB). C57BL/6J (11 weeks old; 30 g) were anesthetized by the intraperitoneal injection of tribromoethanol (320 mg kg^−1^, i.p.) and positioned in a stereotaxic apparatus. Mice were injected with 1 µL of APP‐C31 (200 µm), 1 µL of Aβ_40_ peptide (100 µm) or 1 µL of the mixture of Aβ_40_ (100 µm) and APP‐C31 (200 µm) peptides into the hippocampus (anteroposterior, −2.0 mm; mediolateral, ±1.5 mm; dorsoventral, −1.7 mm from bregma) according to the mouse brain atlas.^[^ ^82^ ^]^ All infusions were given using a Hamilton syringe equipped with a 26‐gauge beveled needle and attached to a Nanojet stereotaxic syringe pump (Chemyx, Inc., TX, USA). Infusions were delivered at a rate of 0.2  µL min^−1^. After injection, the needle was left in place for an additional 5 min before being slowly retracted. After 10 days, animals were sacrificed and their brains were harvested after surgery.

### Immunohistochemistry

Mice were perfused with phosphate‐buffered saline (GIBCO) and 4% paraformaldehyde (PFA) solution, and then the brains were post‐fixed with 4% PFA overnight and dehydrated with 30% sucrose solution. The tissue samples were embedded in Tissue‐Tek OCT compound (Sakura Finetek, Tokyo, Japan) and then cryosectioned at 35 µm thickness using a cryotome (CM1520, Leica, Wetzlar, Austria). Immunofluorescence staining was performed as previously described^[^ ^83^ ^]^ using anti‐Aβ antibody (6E10; 1:1,000), anti‐APP antibody (Y188; 1:500), anti‐Iba1 antibody (1:1,000, Wako Life Sciences Inc., Japan), an appropriate fluorescence‐conjugated secondary antibody, and Hoechst 33 258 (1:10,000; Life Technologies, CA, USA) for counterstaining. The images were obtained using a fluorescent microscope (Leica DMI4000 B, Leica, Wetzlar, Germany).

### ITC

All samples were dissolved in the buffered solution (20 mm HEPES, pH 7.4), and the resultant solutions were degassed for 3 min prior to the loading into the ITC instrument. To investigate the binding affinity of APP‐C31 to Aβ_40_, the final concentrations of APP‐C31 (in the syringe) and Aβ_40_ (in the cell) were adjusted to 600 and 30 µm, respectively. To measure the binding affinity of metal ions to APP‐C31, 2.1 mm of CuCl_2_ or ZnCl_2_ (in the syringe) and 100 µm of APP‐C31 (in the cell) were used for experiments. Titration experiments composed of 28 injections were conducted. The injection volume was 2 µL for the first injection to minimize effects of bubbles and 10 µL for the remaining injections. To prevent Aβ_40_ aggregation, the temperature and stirring speed were set to 10 °C and 260 rpm, respectively. The initial delay was 1800 s and the reference power was 10 µcal s^−1^. The heats of dilution of APP‐C31, CuCl_2_, and ZnCl_2_ titrated into the same buffer were also examined. The ITC thermogram and binding isotherm were displayed after subtracting the dilution heat. The binding isotherms after baseline correction were fitted to the one set of sites binding model incorporated in the MicroCal Origin 7.0 software.^[^ ^84^ ^]^

### 2D ^1^H–^15^N HSQC NMR Spectroscopy

NMR samples were prepared as described in the previous study.^[^ ^66^ ^]^ Uniformly ^15^N‐labeled Aβ_40_ (rPeptide Inc., Bogart, GA, USA) was initially dissolved in NH_4_OH (1% w/w, aq; 10 µL). After lyophilizing the resulting solution, the peptide was stored at −80 °C. A solution of the ^15^N‐labeled Aβ_40_ monomer was prepared by dissolving the lyophilized peptide in the chilled NaOH solution (10 mm) to make a stock concentration of ≈200 µm. For 2D ^1^H–^15^N HSQC measurements, the stock solution was further diluted to be 40 µm of ^15^N‐labeled Aβ_40_ in the buffered solution (20 mm HEPES, pH 7.4) containing 10% v/v D_2_O, and then treated with APP‐C31 (200 µm). All HSQC spectra were obtained at 5 °C. Each spectrum was obtained from 256 *t*_1_ experiments using 64 transients and 1 s recycle delay. Data were processed by NMRPipe^[^ ^85^ ^]^ and analyzed by Sparky.^[^ ^86^ ^]^ The assignment of backbone resonance was carried out based on the previously assigned information.^[^ ^84^ ^]^ CSP (Δ*δ*_NH_) was calculated by the Equation (3):

(3) $$\Delta \;\delta_{\mathrm{NH}}=\sqrt{\Delta \delta_{H}^{2}+\;{\left(\frac{\Delta \delta_{N}}{6.5}\right)}^{2}}\;$$

where Δ*δ*_H_ and Δ*δ*_N_ represent the change of chemical shift in the proton and nitrogen dimensions, respectively.

### NMR Studies with Aβ Fibrils

The solution of fibrillar Aβ_40_ was prepared by incubation of freshly prepared Aβ_40_ (200 µm) for 40 h at 37 °C with constant agitation (250 rpm) in 20 mm HEPES, pH 7.4. Aβ_40_ fibrils were further diluted to be 100 µm in the buffered solution (20 mm HEPES, pH 7.4) containing 10% v/v D_2_O and then added with APP‐C31 (20 µm). A standard pulse sequence with water suppression by excitation sculpting (zgesgp from Bruker Topspin using the standard parameter set) was used. For each spectrum, 128 transients were collected into 32,768 data points over a spectrum width of 20 ppm. Data were analyzed using TopSpin 3.6.1 software.^[^ ^87^ ^]^

### Preparation of MD Simulations

The atomistic NMR structure of Aβ_40_ deposited in the protein data bank (PDB 2LFM)^[^ ^55^ ^]^ was used as the initial conformation of Aβ monomer. For APP‐C31, the X‐ray crystal structure of APP_666‐693_ excised from the intracellular domain of APP complexed with Fe65 protein (PDB 3DXC)^[^ ^56^ ^]^ was selected as the starting conformation of the protein. MD simulations of APP_666–693_ monomer, Aβ_40_ monomer, and the APP_666–693_–Aβ_40_ dimer were conducted by a GPU enabled Amber16 software suite.^[^ ^47^ ^]^ Each monomer was immersed in an octahedral solvation box filled with TIP3P water molecules.^[^ ^48^ ^]^ For the APP_666–693_–Aβ_40_ dimer, 34 distinct dimeric conformations that were sampled from metadynamics MD simulations were used as the initial conformations (vide infra). The minimal distance from the protein surface to the solvation boundary was at least 12 Å. Monovalent counterions (Na^+^ or Cl^–^) were added to ensure the charge neutrality of the simulated systems. To model the peptides, the ff14SB all atom amino acid force fields parameter set was adopted.^[^ ^49^ ^]^ MD simulations were applied to 0.2 fs time integration steps in conjunction with the Settle algorithm that fixes the bond distance between a hydrogen atom and its heavy atom partner.^[^ ^50^ ^]^ Particle Mesh Ewald (PME) method was employed to accelerate the calculations of long‐range electrostatic interactions.^[^ ^88^ ^]^ The simulations began by 4,000 steps of energy minimizations. The temperature of each solvated model was increased from 0 to 100 K in 2 ns while restraining each carbon atom with a harmonic potential of spring constant as 2 kcal mol^−1^ Å^−2^. The temperature was then increased from 100 to 300 K in 10 ns while restraining each Cα atom with a harmonic potential of spring constant as 10 kcal mol^−1^ Å^−2^. Prior to the production periods, additional 10 ns of equilibrations was performed while keeping the temperature at 300 K and the pressure at 1 atm. The production runs lasted for 500 ns for the monomer and dimer models without any restraints to the solutes.

### Metadynamics MD Simulations

Metadynamics MD simulations were employed to investigate the interfaces of the APP_666–693_–Aβ_40_ dimer. The COM distance of the two monomeric peptides, APP_666–693_ and Aβ_40_, was used as the reaction coordinate of the dimerization. Gibbs free energy profile was computed along the chosen reaction coordinates without additional processing of the simulation data.^[^ ^46^ ^]^ Using the metadynamics MD simulations, reversible association of the two monomers was monitored followed by achieving an enriched set of the associated dimer conformations. Metadynamics MD simulations were initiated from the last step of the constant temperature‐pressure MD simulations of the APP_666–693_–Aβ_40_ dimer. To enhance the convergence of the simulations, the well‐tempered metadynamics method was applied with the bias temperature of 500 K.^[^ ^51^, ^52^ ^]^ The multi‐walker method was used to diversify the dimer conformations.^[^ ^53^ ^]^ Total 12 replicas of the dimers were simulated. Each replica was simulated for 700 ns resulting in 8.4 µs of gross simulation time. The COM distance was sampled from 5 to 40 Å and was monitored by the well‐tempered metadynamics algorithm. Specifically, the 35 Å interval was discretized with equally spaced windows of 0.5 Å. As the simulation samples a certain window, the algorithm incremented the height of bias potential of the window, which promoted the simulations to sample less explored COM distance within the interval. Initially, every visitation of a certain window increased the bias potential by 0.005 kcal mol^−1^, and the height of the increment gradually reduced with the help of the well‐tempered metadynamics algorithm. The simulation temperature was held constant at 300 K. The convergence of the metadynamics MD simulations was identified by observing the Gibbs free energy difference (ΔG) of the bound and unbound states of the dimer. The averaged potential of mean‐force (PMF) from 35 to 40 Å was determined as the energy of the unbound state, whereas that of the minimum was treated as the energy of the bound state. The energy difference began to converge after 7 µs in gross simulation time. Reversible binding and unbinding events were observed from individual metadynamics MD trajectories (Replicas A, D, H, K, and L). These observations suggested that the metadynamics MD trajectories successfully explored the underlying conformation space of the APP_666‐693_–Aβ_40_ dimer interface. GPU enabled NAMD v2.12 software was used for the metadynamics MD simulations.^[^ ^89^ ^]^

### Structural Clustering of the Dimer Interfaces Sampled from Metadynamics MD Simulations

Upon completion of metadynamics MD simulations, structural cluster analysis was carried out to identify the distinct APP_666–693_–Aβ_40_ dimer interfaces. First, the dimer interfaces that show the COM distances between 10 and 15 Å were collected, where the computed Gibbs free energy profile was minimized. Those collected interfaces were subject to the structural clustering analysis using VMD software.^[^ ^90^ ^]^ Two conformers having the backbone RMSD less than 3.0 Å were considered to be similar structures, which resulted in 34 different structural clusters. A representative conformation of each structural cluster was subject to additional 500 ns MD simulations without structural constraints. Upon completion of the constant temperature MD simulations, the COM distance between APP_666–693_ and Aβ_40_ was observed. 12 of the selected interfaces showed a stable COM distance profile through the course of MD simulation. The number of intermolecular contacts between APP_666–693_ and the Arg5, Glu11, His13, Gln15, Leu17, Phe20, and Ala21 residues in Aβ_40_ whose CSPs were pronounced in the 2D ^1^H–^15^N HSQC NMR experiments was investigated. The number of intermolecular contacts was defined as the number of heavy atoms of APP_666‐693_ within 10.0 Å of the backbone nitrogen atoms of the seven amino acid residues in Aβ_40_. Upon thorough inspection, Models 7 and 19 that showed the relatively stable COM distance and the intermolecular contacts congruent with the 2D ^1^H–^15^N HSQC NMR studies were selected as the two representative models of the APP_666–693_–Aβ_40_ dimer interfaces and labeled as Model I and II, respectively.

### Statistical Analysis

All statistical analyses were performed using Origin software.^[^ ^91^ ^]^ The comparison between the two groups was performed with Student's *t*‐test. Values were denoted as mean ± s.e.m. (standard error of the mean). Statistical difference was considered significant at *p < 0.05, **p < 0.01, and ***p < 0.001. All the ThT assays were performed in triplicate. For the TEM analysis, the location of samples on the grids was randomly selected for taking more than 15 images per each grid. For the cell viability assay with microinjection, at least 100 cells from each sample were injected, and the experiments were performed at least in triplicate. The size of ^HF647^Aβ_40_ aggregates was determined by the “analyze particles” program of Fiji software based on the previously reported studies.^[^ ^37^ ^]^ All pixels above the threshold value were selected to calculate the area of ^HF647^Aβ_40_ aggregates. The analysis included ^HF647^Aβ_40_ aggregates with the size larger than 0.05 µm^2^. The neurite of a neuron measured from the soma to the tip was analyzed using the Neuron J program of Fiji software.^[^ ^81^ ^]^ At least five randomly selected fields were captured with an Olympus IX71 microscope.

## Conflict of Interest

The authors declare no conflict of interest.

## Author Contributions

E.N. and M.H.L. designed the research. E.N. performed the ThT, ESI–MS, CD, TEM, and biochemical and cell studies with data analyses. Y.L. and Y.‐H.L. conducted ^1^H NMR, 2D ^1^H–^15^N HSQC NMR, and ITC experiments and analyzed the data. J.Y.P. and M.‐H.B. carried out MD simulations with analysis. H.D., M.K., and J.H. (Jinju Han) contributed to the toxicity studies using human neurons. B.J., S.P., and D.Y.L conducted in vivo experiments and analyzed the data. J.H. (Jiyeon Han) performed the ESI–MS experiments with data analysis. E.N., J.H. (Jiyeon Han), M.‐H.B., and M.H.L. wrote the manuscript with input from all authors.

## Supporting information

Supporting Information

## Data Availability

The data that support the findings of this study are available from the corresponding author upon reasonable request.
