# Supplementary material for: APP‐C31: An Intracellular Promoter of Both Metal‐Free and Metal‐Bound Amyloid‐β40 Aggregation and Toxicity in Alzheimer's Disease
Source: Adv Sci (Weinh). 2023 Nov 10;11(4):2307182. doi: 10.1002/advs.202307182 (PMC10811509; doi:10.1002/advs.202307182)
Supplement: Supplementary file 1 — Supporting Information [file ADVS-11-2307182-s001.pdf]

ADVANCED SCIENCE
Open Access

Supporting Information

for *Adv. Sci.*, DOI 10.1002/advs.202307182

APP-C31: An Intracellular Promoter of Both Metal-Free and Metal-Bound Amyloid-β_{40} Aggregation and Toxicity in Alzheimer's Disease

Eunju Nam, Yuxi Lin, Jiyong Park, Hyunsu Do, Jiyeon Han, Bohyeon Jeong, Subin Park, Da Yong Lee, Mingeun Kim, Jinju Han, Mu-Hyun Baik*, Young-Ho Lee* and Mi Hee Lim**

Supporting Information

APP-C31: An Intracellular Promoter of Both Metal-Free and Metal-Bound Amyloid-β_{40} Aggregation and Toxicity in Alzheimer's Disease

Eunju Nam, Yuxi Lin, Jiyong Park, Hyunsu Do, Jiyeon Han, Bohyeon Jeong, Subin Park, Da Yong Lee, Mingeun Kim, Jinju Han,* Mu-Hyun Baik,* Young-Ho Lee,* and Mi Hee Lim*

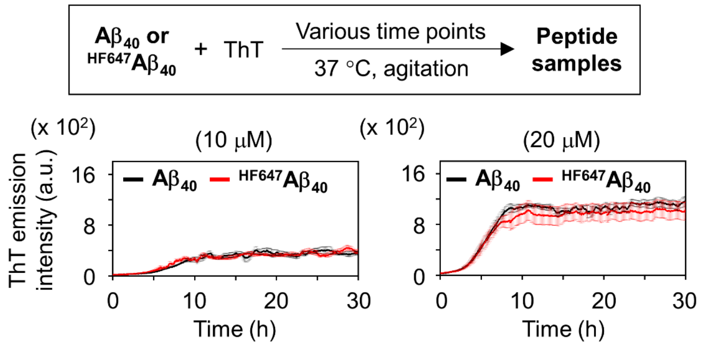

Figure S1. Aggregation kinetics of Aβ_{40} and ^{HF647}Aβ_{40} monitored by the ThT assay. It should be noted that Aβ_{40} only used in this experiment was obtained from the same manufacturer as ^{HF647}Aβ_{40} (Anaspec), taking into account the distinct aggregation kinetics of Aβ depending on its commercial source. Experiments were performed in quintuplicate. Conditions: [Aβ_{40} and ^{HF647}Aβ_{40}] = 10 or 20 μM; 20 mM HEPES, pH 7.4, 150 mM NaCl; 37 °C; quiescent conditions.

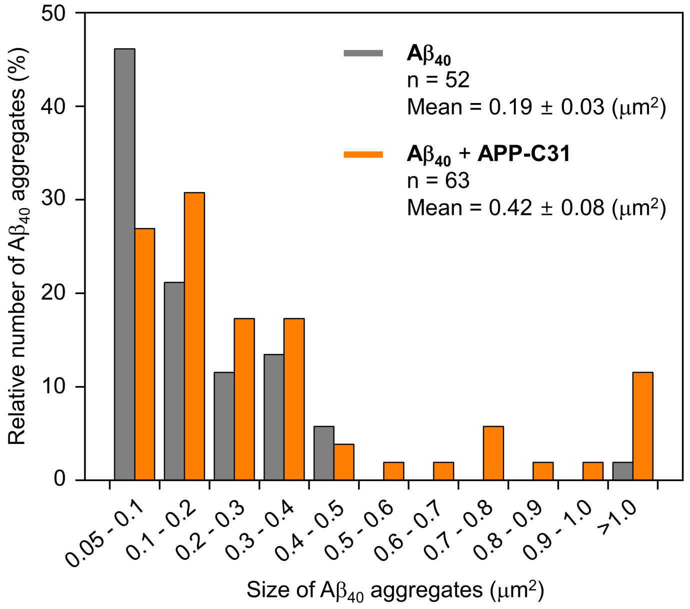

Figure S2. Size distribution of intracellular Aβ_{40} aggregates produced in the absence and presence of APP-C31. The relative number of Aβ aggregates was calculated by dividing the value in each bin by the total number of aggregates. Conditions: [APP-C31] = 10 μM; [^{HF647}Aβ_{40}] = 10 μM; 24 h incubation; n = 52 for the group of ^{HF647}Aβ_{40}; n = 63 for the group of ^{HF647}Aβ_{40} with APP-C31. All pixels above the threshold value were selected to calculate the area of ^{HF647}Aβ_{40} aggregates.

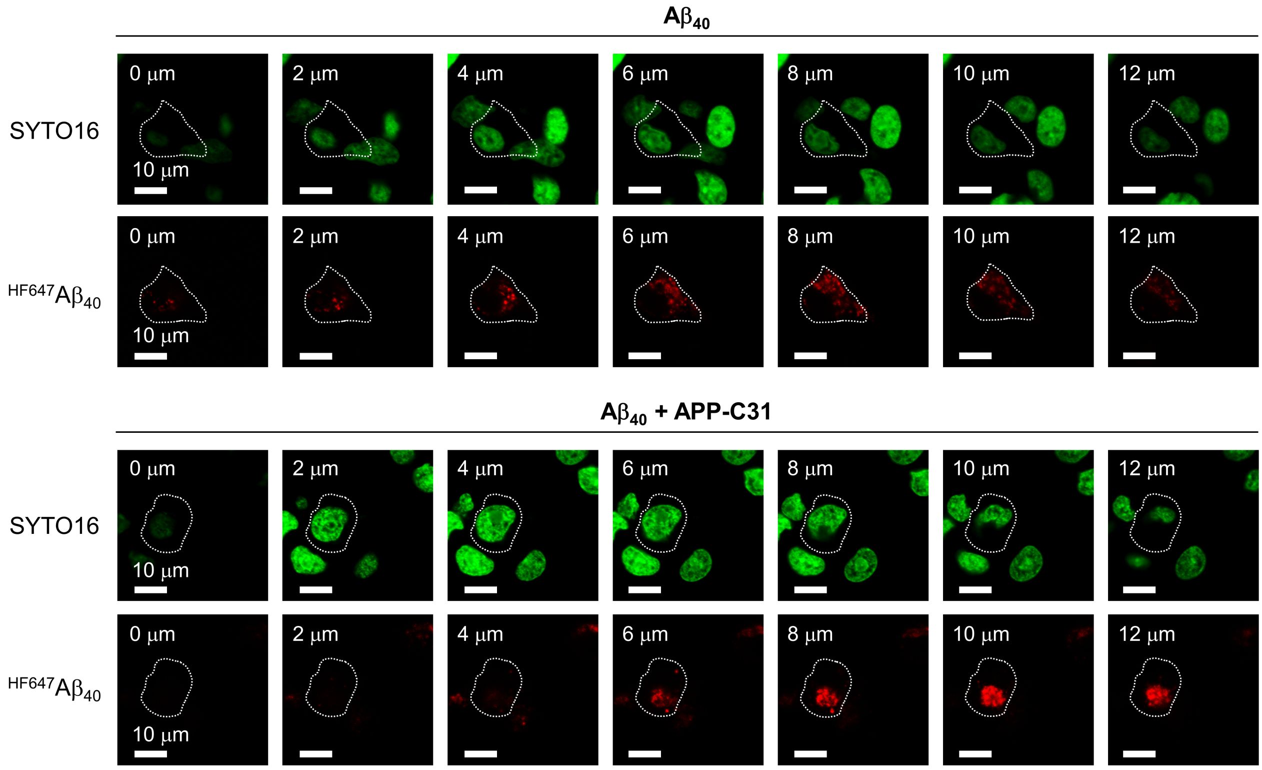

Figure S3. Individual green and red fluorescence images of the cells treated with $^{HF647}A\beta_{40}$ in the absence and presence of APP-C31. Conditions: [$^{HF647}A\beta_{40}$] = 10 μM; [APP-C31] = 10 μM; [SYTO16] = 2.5 μM; 24 h incubation. Scale bar = 10 μm.

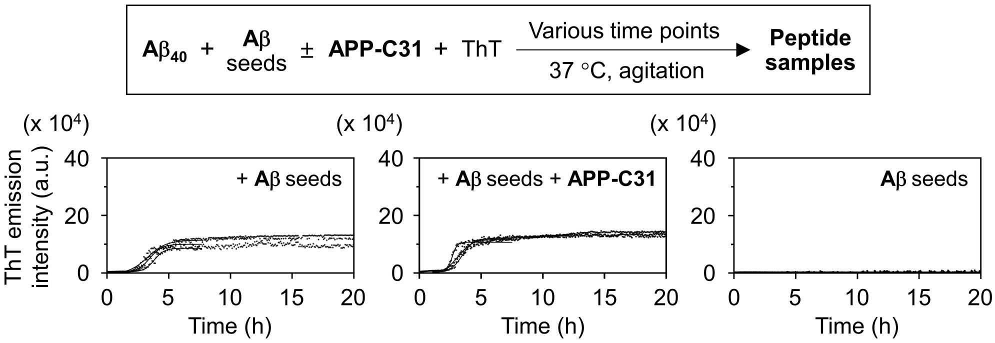

Figure S4. Impact of APP-C31 on the aggregation of $A\beta_{40}$ in the presence of $A\beta_{40}$ seeds. The aggregation of $A\beta_{40}$ upon treatment of $A\beta_{40}$ seeds in the absence and presence of APP-C31 (1 equiv) was monitored by the ThT assay. Experiments were performed in triplicate. Conditions: [$A\beta_{40}$] = 20 μM; [APP-C31] = 20 μM; $A\beta_{40}$ seeds (5% v/v); 20 mM HEPES, pH 7.4, 150 mM NaCl; 37 °C; constant agitation (559 cpm).

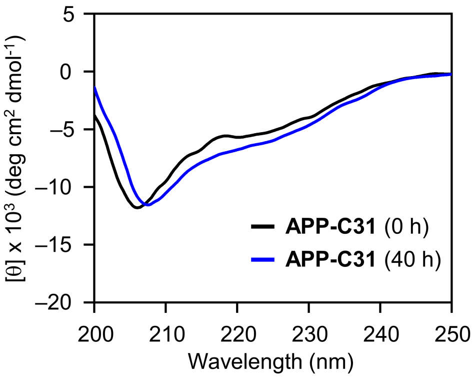

Figure S5. Secondary structure of APP-C31 observed by CD spectroscopy. The CD spectra of APP-C31 were obtained after 0 h and 40 h incubation. The far-UV circular dichroism (CD) spectrum of APP-C31 manifested a typical pattern of a 3_{10} helix showing the local minimum at *ca*. 207 nm and 222 nm.[1,2] Conditions: [APP-C31] = 100 μM; 20 mM HEPES, pH 7.4, 150 mM NaF; 37 °C. The measurements were conducted in triplicate.

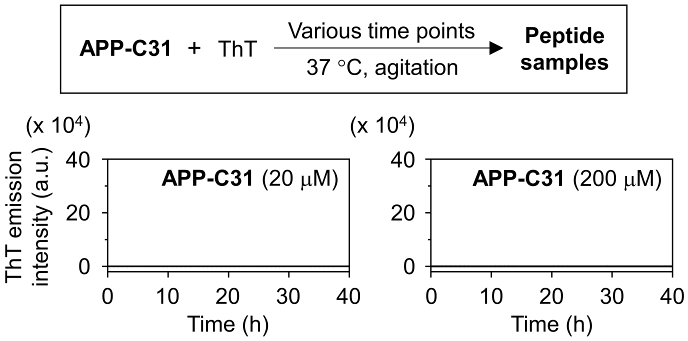

Figure S6. Aggregation kinetics of APP-C31 monitored by the ThT assay. Conditions: [APP-C31] = 20 or 200 μM; 20 mM HEPES, pH 7.4, 150 mM NaCl; 37 °C; constant agitation (559 cpm). Experiments were carried out in triplicate.

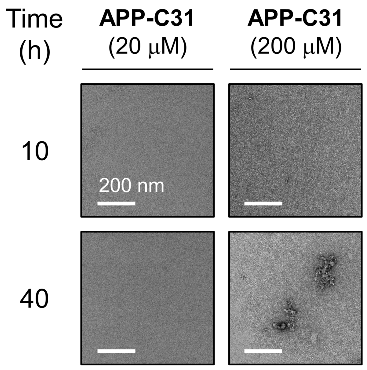

Figure S7. Morphology of APP-C31 detected by TEM. Conditions: [APP-C31] = 20 or 200 μM; 20 mM HEPES, pH 7.4, 150 mM NaCl; 40 h; 37 °C; constant agitation (559 cpm).

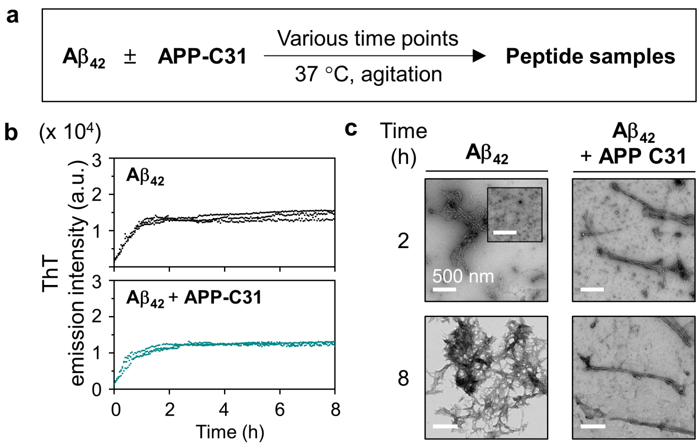

Figure S8. Impact of APP-C31 on the aggregation of Aβ_{42}. a) Scheme of the aggregation experiments. b) Aggregation kinetics of Aβ_{42} in the absence and presence of APP-C31 analyzed by the ThT assay. Experiments were conducted in triplicate. Conditions: [Aβ_{42}] = 20 μM; [APP-C31] = 20 μM; 20 mM HEPES, pH 7.4, 150 mM NaCl; 37 °C; Constant agitation (559 cpm). c) Morphology of the resultant Aβ_{42} aggregates with or without APP-C31 detected by TEM. Scale bar = 500 nm. Conditions: [Aβ_{42}] = 25 μM; [APP-C31] = 25 μM; 20 mM HEPES, pH 7.4, 150 mM NaCl; 37 ºC; constant agitation.

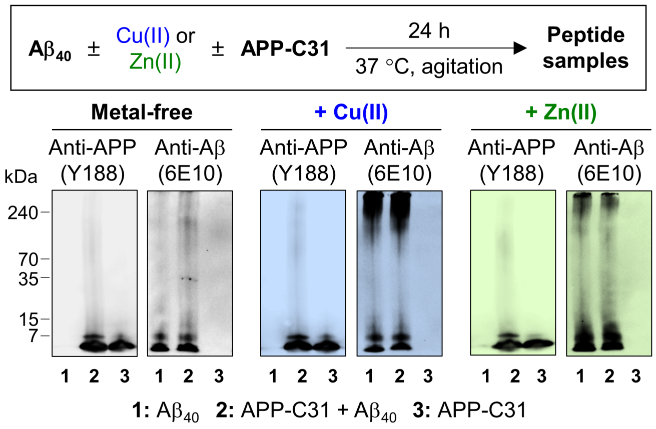

Figure S9. Analysis of the size distribution of the resultant APP-C31 and Aβ_{40} species. The size distribution of the resultant APP-C31 and Aβ_{40} species was visualized through gel/Western blots with an anti-APP *C*-terminus antibody (Y188) and an anti-Aβ antibody (6E10), respectively. Conditions: [APP-C31] = 25 μM; [Aβ_{40}] = 25 μM; [$CuCl_2$] = 25 μM; [$ZnCl_2$] = 25 μM; 20 mM HEPES, pH 7.4, 150 mM NaCl; 37 °C; constant agitation (250 rpm).

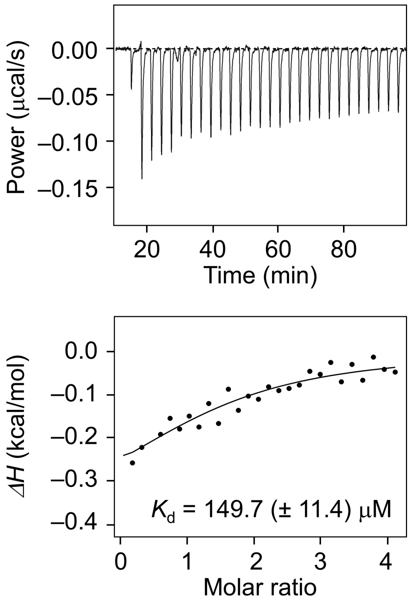

Figure S10. Interaction of $A\beta_{40}$ with APP-C31 monitored by ITC. The ITC thermogram (upper panel) and binding isotherm (lower panel) were obtained upon titration of APP-C31 into $A\beta_{40}$. The K_d value was calculated from three independent measurements. Conditions: [$A\beta_{40}$] = 30 μM; [APP-C31] = 600 μM; 20 mM HEPES, pH 7.4; 10 °C.

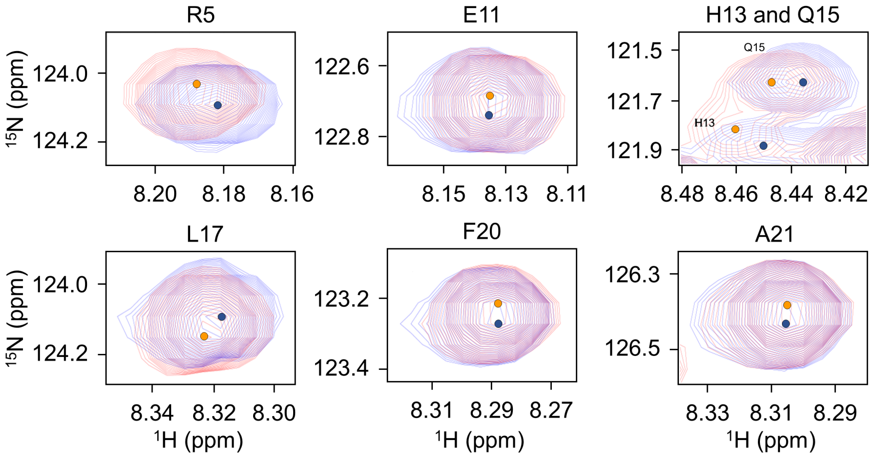

Figure S11. Zoomed-in images of the 2D ^{1}H-^{15}N HSQC NMR spectra obtained from the samples of Aβ_{40} with and without APP-C31 The spectra corresponding to Arg5, Glu11, His13, Gln15, Leu17, Phe20, and Ala21 of ^{15}N-labeled Aβ_{40} are presented. Conditions: [^{15}N-labeled Aβ_{40}] = 40 μM; [APP-C31] = 200 μM; 20 mM HEPES, pH 7.4; 5 °C.

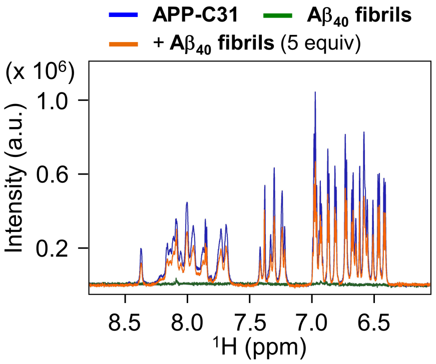

Figure S12. Interaction between APP-C31 and amyloid fibrils of $A\beta_{40}$ monitored by ^{1}H NMR (800 MHz). Conditions: [$A\beta_{40}$ fibrils] = 100 μM; [APP-C31] = 20 μM; 20 mM HEPES, pH 7.4; 5 °C.

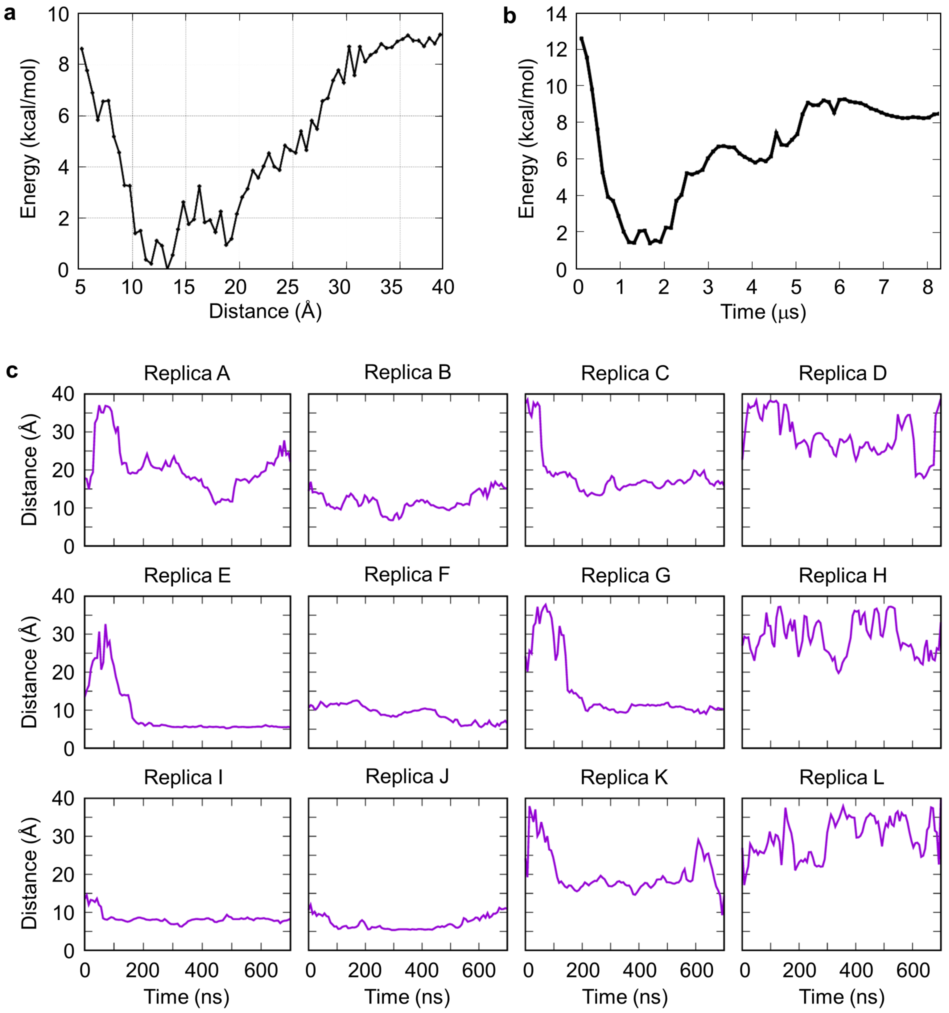

Figure S13. Gibbs free energy profile and COM distance between $APP_{666\text{-}693}$ and $A\beta_{40}$ measured from metadynamics MD simulations. a) Gibbs free energy of profile of the dimer interface as a function of the COM distance between $APP_{666\text{-}693}$ and $A\beta_{40}$. b) Convergence of metadynamics MD simulations monitored by observing the Gibbs free energy difference (ΔG) of the bound and unbound states of the dimer. c) COM distance sampled from each replica of metadynamics MD simulations of the $APP_{666\text{-}693}$–$A\beta_{40}$ dimer. The X-axis is the gross simulation time that is the simulation time of each replica multiplied by the number of replicas. Reversible binding and unbinding events observed from individual metadynamics MD trajectories (Replicas A, D, H, K, and L) suggested that our metadynamics MD trajectories successfully explored the underlying conformation space of the $APP_{666\text{-}693}$–$A\beta_{40}$ dimer interface.

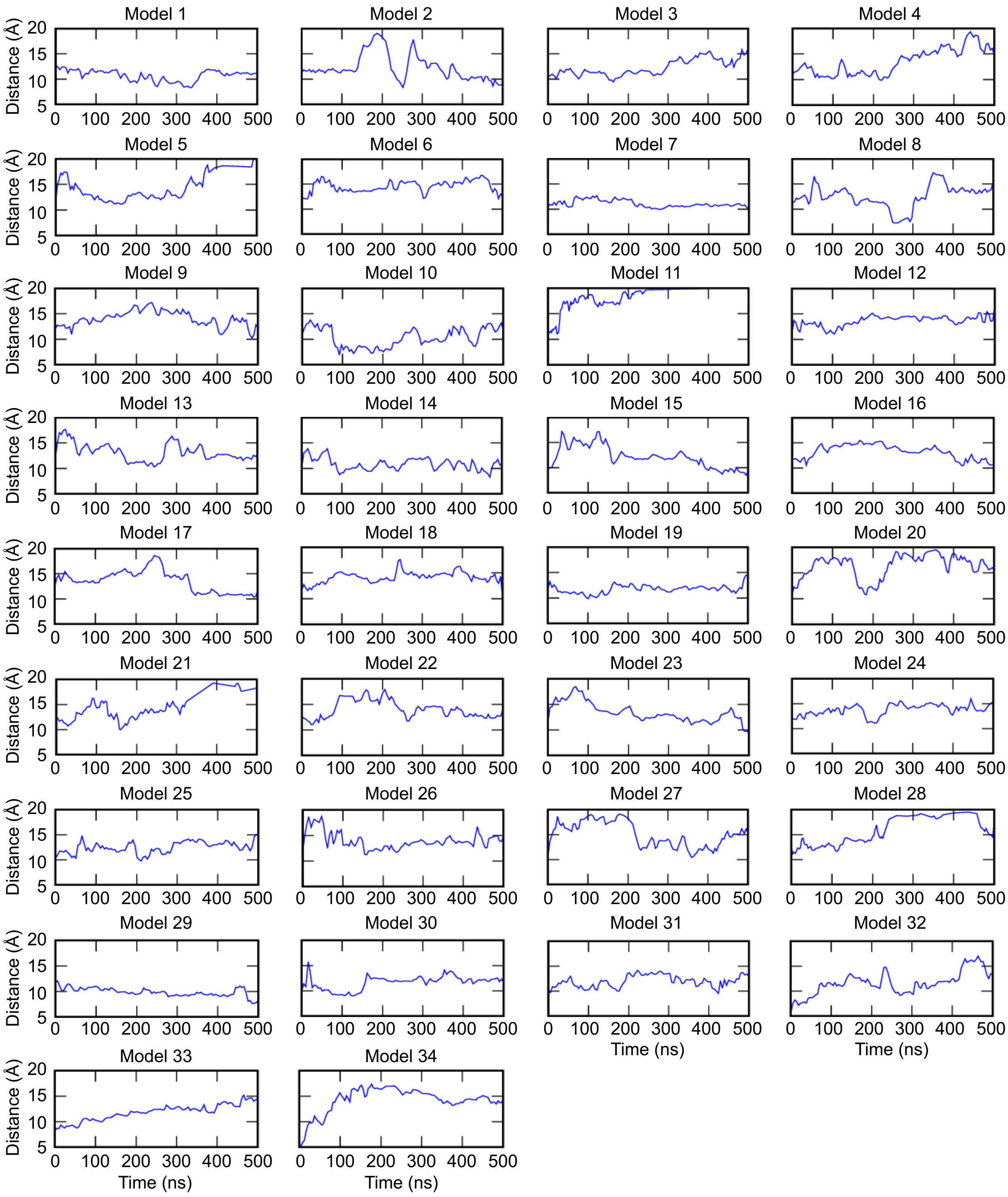

Figure S14. COM distance from the unbiased MD simulations of $APP_{666\text{-}693}$–$A\beta_{40}$ dimer interfaces.

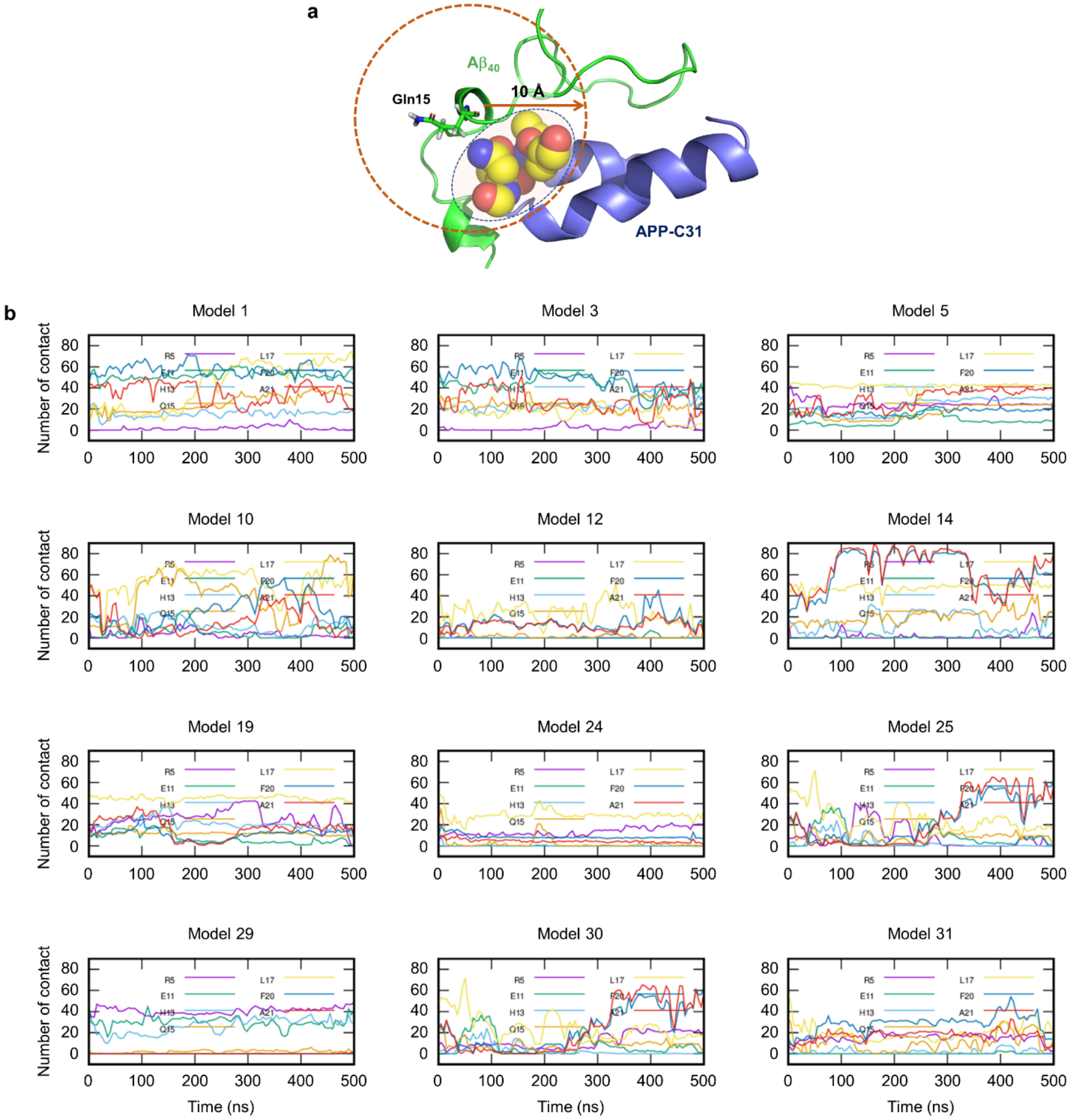

Figure S15. Intermolecular contacts between $APP_{666\text{-}693}$ and the Arg5, Glu11, His13, Gln15, Leu17, Phe20, or Ala21 residues in $A\beta_{40}$. a) A figure for the definition of intermolecular contacts. The number of intermolecular contacts was defined as the number of heavy atoms of $APP_{666\text{-}693}$ within 10.0 Å of the backbone nitrogen atoms of the seven amino acid residues in $A\beta_{40}$ that exhibited the noticeable chemical shift perturbations observed by 2D ^{1}H–^{15}N HSQC NMR. The heavy atoms that meet the criteria were presented in the space-filling representation. b) Number of heavy atoms in $APP_{666\text{-}693}$ that simultaneously interact with the Arg5, Glu11, His13, Gln15, Leu17, Phe20, or Ala21 residues in $A\beta_{40}$.

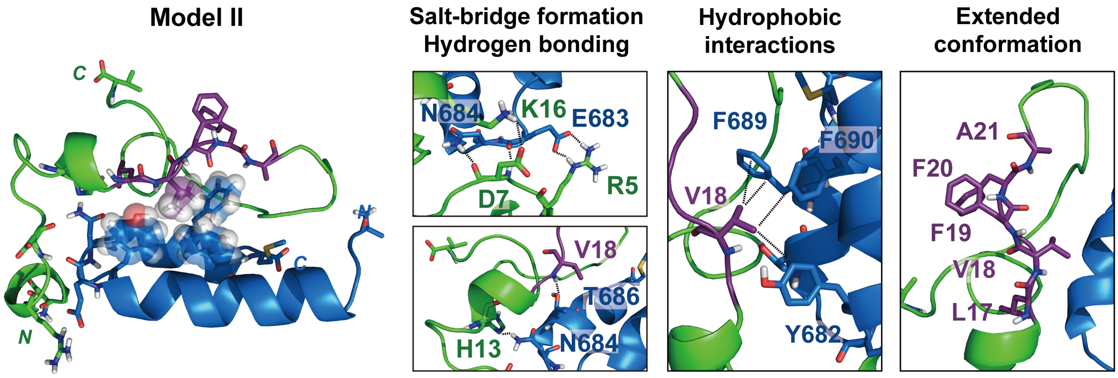

Figure S16. Representative model (Model II) of the $APP_{666\text{-}693}$–$A\beta_{40}$ interfaces from the trajectories of MD simulations. Possible hydrogen bonds within 3.0 Å and hydrophobic interactions observed within 4.0 Å are indicated with dashed black lines. The amino acid residues involved in hydrophobic interactions are presented in the space-filling representation.

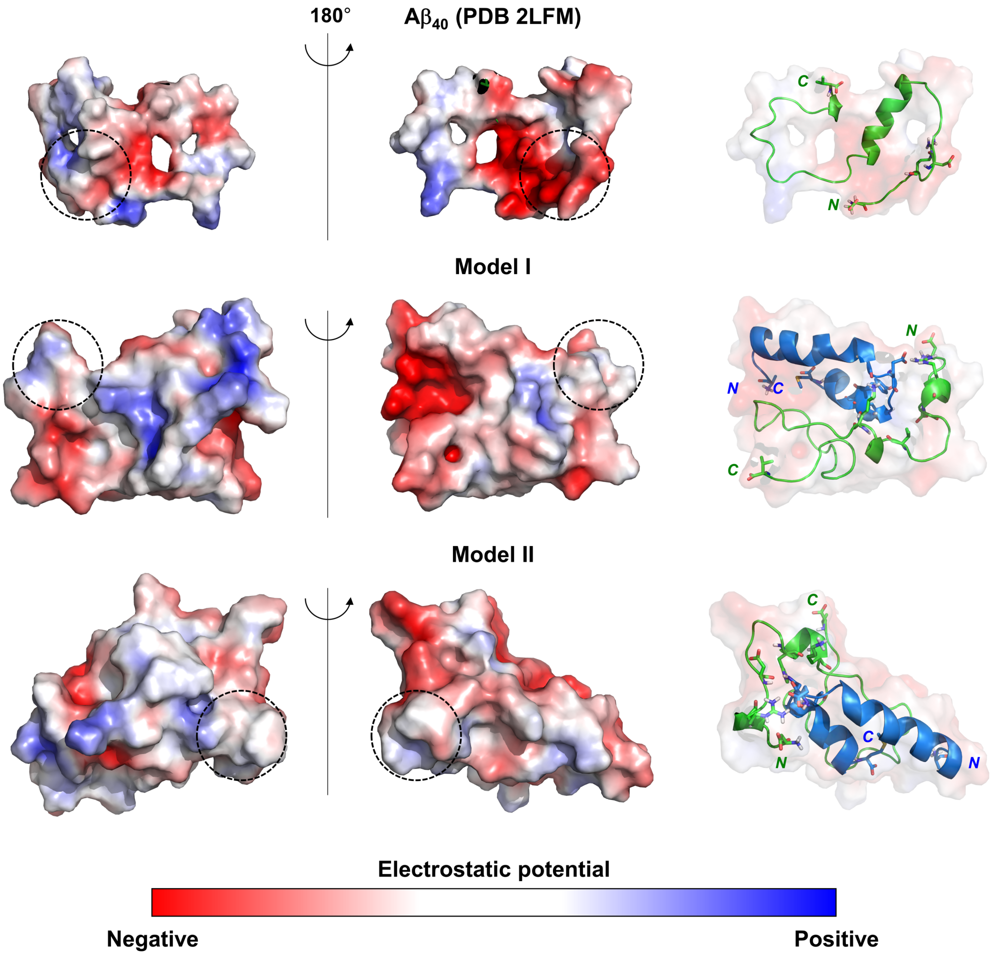

Figure S17. Electrostatic potential mapping on the surface of Aβ_{40} and the simulated Aβ_{40}–APP-C31 complexes. The surface of peptides is presented according to their electrostatic potentials from red (–3 kT/e, negatively charged) to blue (+3 kT/e, positively charged) using PyMoL.[3-5]

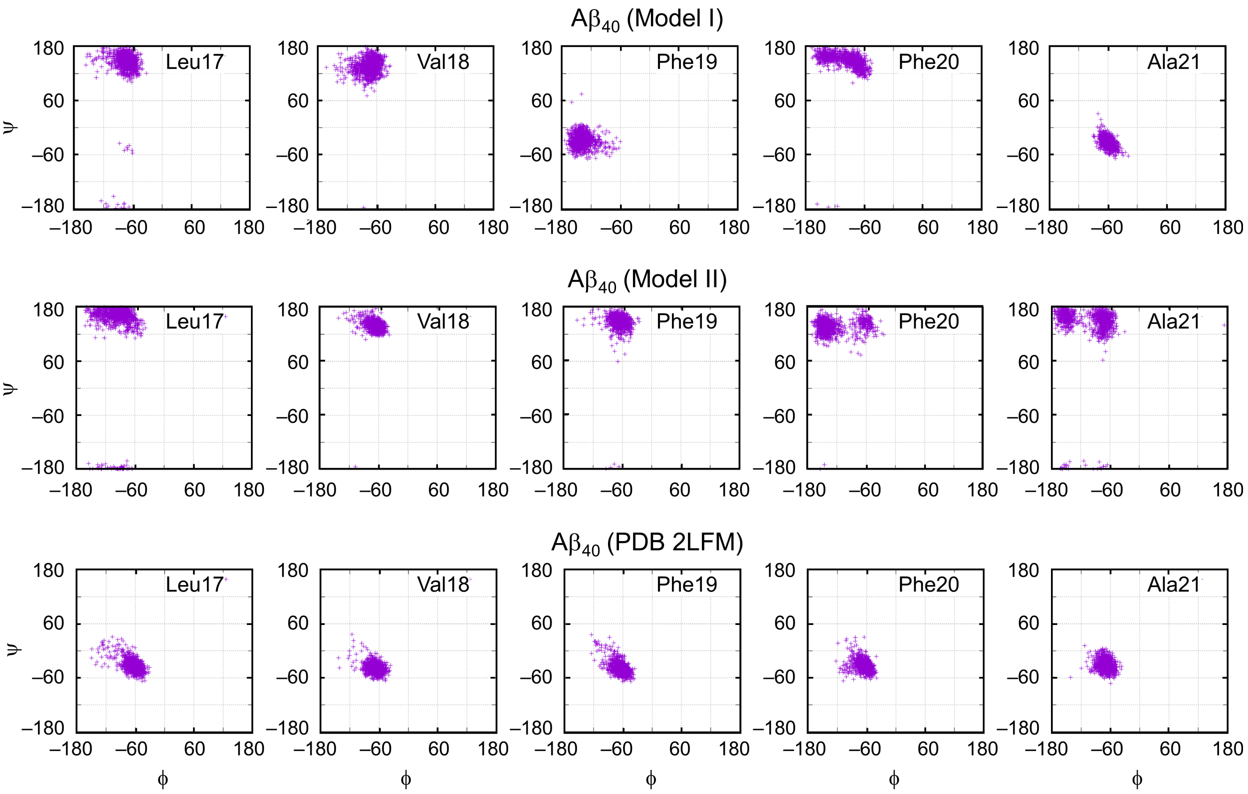

Figure S18. Ramachandran plots for the amino acid residues in the self-recognition site of Aβ_{40} after MD simulations. The ϕ and ψ backbone dihedral angles of Leu17, Val18, Phe19, Phe20, and Ala21 in Aβ_{40} were reported.

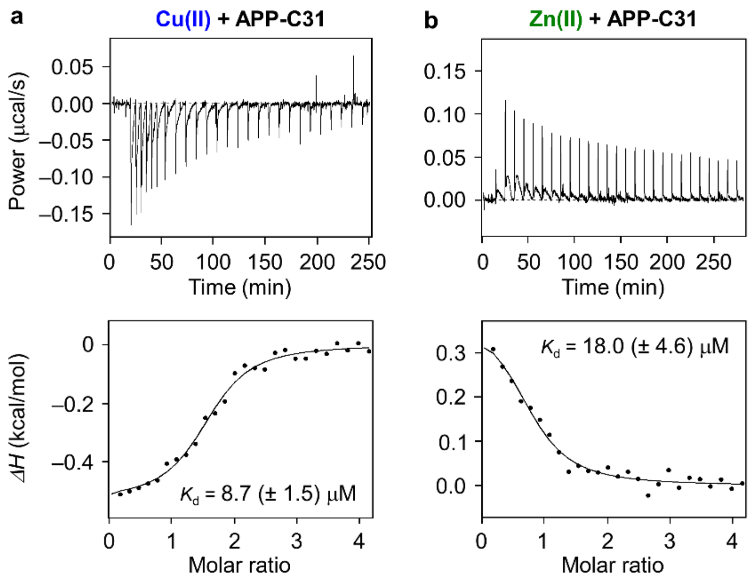

Figures S19. Binding of APP-C31 to Cu(II) or Zn(II) measured by ITC. a,b) The ITC thermograms (upper panel) and binding isotherms (lower panel) obtained upon titrating Cu(II) or Zn(II) into APP-C31 are shown after subtracting the heat of dilution. The error values were calculated from the model fitting. Conditions: [APP-C31] = 100 µM; [$CuCl_2$ or $ZnCl_2$] = 2.1 mM; 20 mM HEPES, pH 7.4; 10 °C.

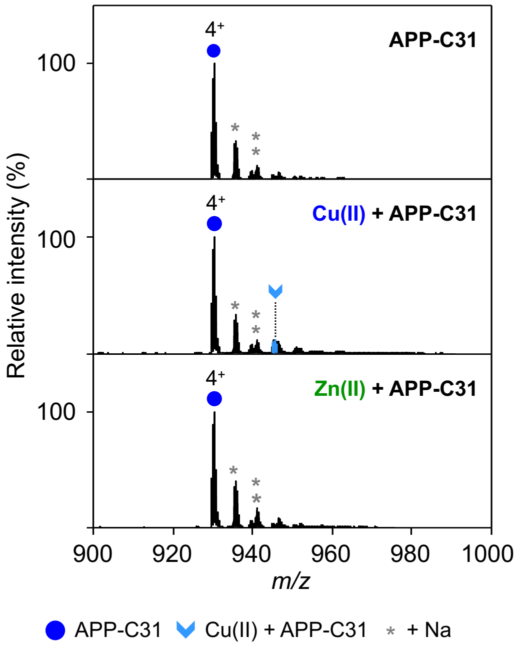

Figure S20. Spectra of APP-C31 treated with Cu(II) or Zn(II) obtained by ESI–MS. Conditions: [APP-C31] = 100 μM; [$CuCl_2$ or $ZnCl_2$] = 100 μM; 20 mM ammonium acetate, pH 7.3; 37 °C; 2 h incubation; no agitation. The samples were diluted by 10 fold prior to injection to the mass spectrometer. Charge states are marked in the MS spectra.

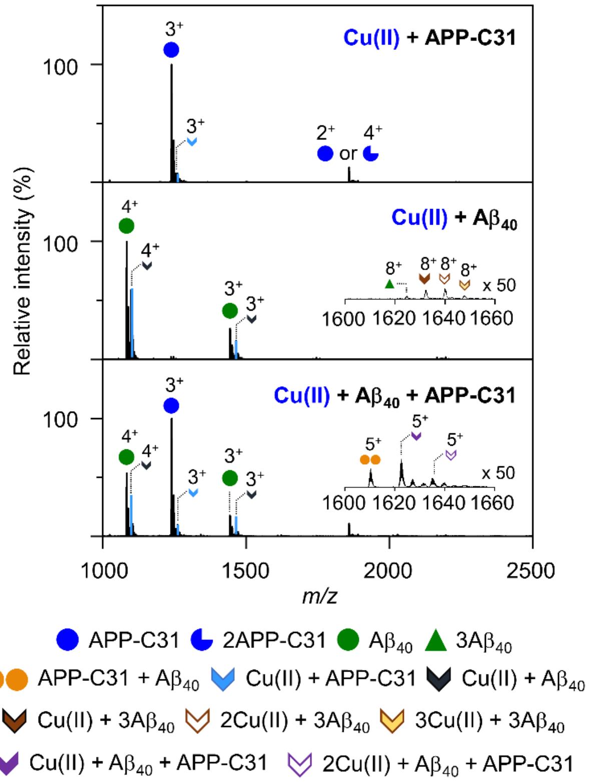

Figure S21. ESI–MS spectra of APP-C31 incubated with $A\beta_{40}$ in the presence of Cu(II). Conditions: [APP-C31] = 100 μM; [$A\beta_{40}$] = 100 μM; [$CuCl_2$] = 100 μM; 20 mM ammonium acetate, pH 7.3; 37 °C; 2 h incubation; no agitation. The samples were diluted by 10 fold prior to injection to the mass spectrometer. Charge states are marked in the MS spectra.

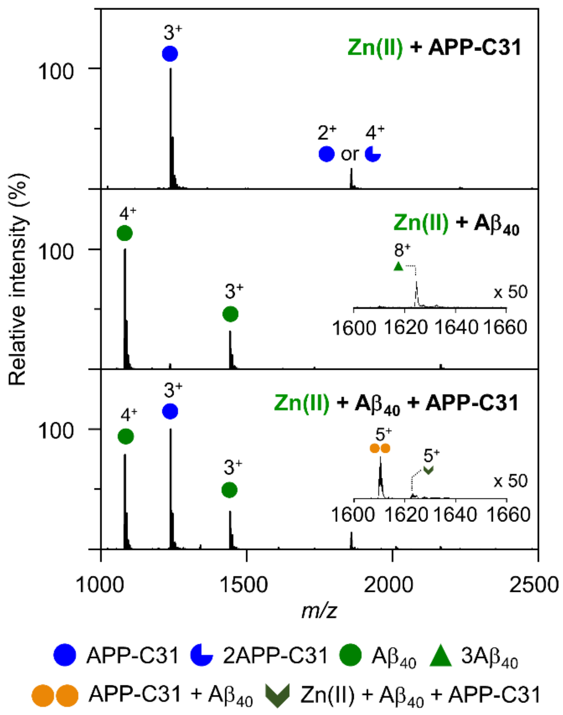

Figure S22. ESI–MS spectra of APP-C31 treated with $A\beta_{40}$ in the presence of Zn(II). Conditions: [APP-C31] = 100 μM; [$A\beta_{40}$] = 100 μM; [$ZnCl_2$] = 100 μM; 20 mM ammonium acetate, pH 7.3; 37 °C; 2 h incubation; no agitation. The samples were diluted by 10 fold prior to injection to the mass spectrometer. Charge states are marked in the MS spectra.

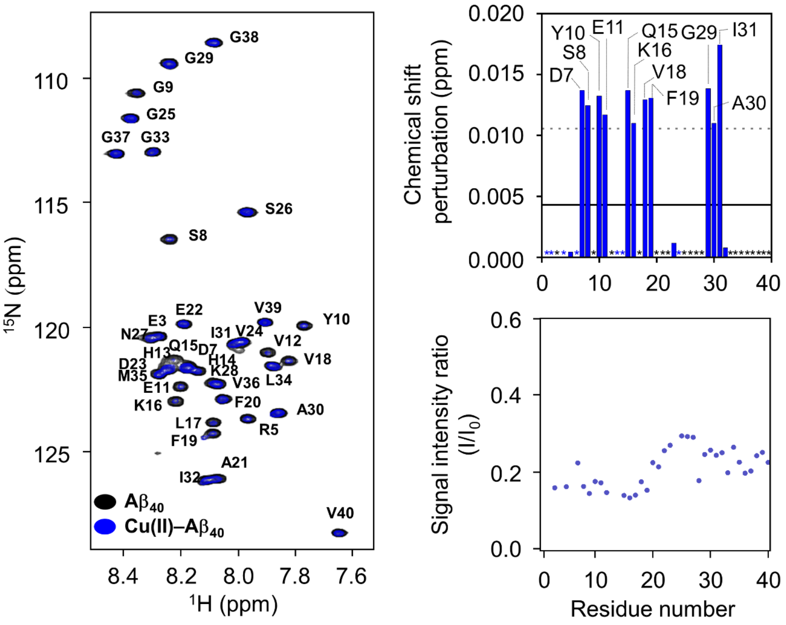

Figure S23. Interaction of Cu(II) and $A\beta_{40}$ analyzed by 2D ^{1}H–^{15}N HSQC NMR spectroscopy (700 MHz). The average of CSPs and the average + one standard deviation are indicated with solid and dashed lines, respectively. It should be noted that the treatment of Cu(II) induced an overall reduction in the signal intensity of ^{15}N-labeled $A\beta_{40}$ due to its paramagnetic effects. Conditions: [^{15}N-labeled $A\beta_{40}$] = 40 μM; [$CuCl_2$] = 20 μM; 20 mM HEPES, pH 7.4; 10 °C. The amino acid residues indicated in blue or black asterisks represent the residues that were unresolved or not significantly shifted, respectively.

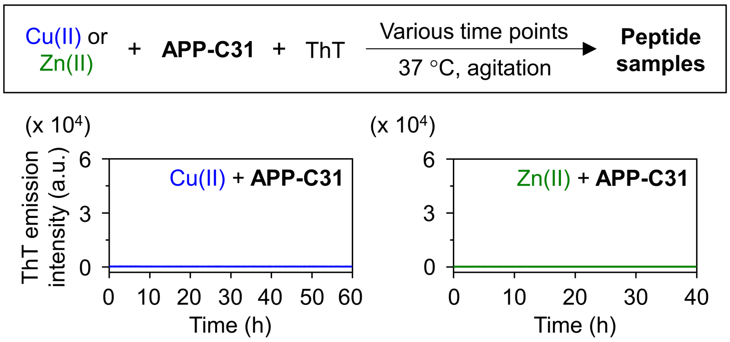

Figure S24. Aggregation kinetics of metal-treated APP-C31 determined by the ThT assay. Experiments were performed in triplicate. Conditions: [APP-C31] = 20 μM; [$CuCl_2$] = 18 μM; [$ZnCl_2$] = 20 μM; 20 mM HEPES, pH 7.4, 150 mM NaCl; 37 °C; constant agitation (559 cpm).

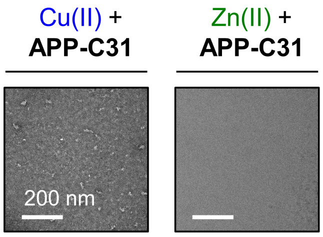

Figure S25. Morphology of metal-treated APP-C31 monitored by TEM. The samples containing APP-C31 were incubated with Cu(II) or Zn(II) for 60 h or 40 h, respectively. Conditions: [APP-C31] = 20 μM; [$CuCl_2$] = 18 μM; [$ZnCl_2$] = 20 μM; 20 mM HEPES, pH 7.4, 150 mM NaCl; 37 °C; constant agitation (559 cpm).

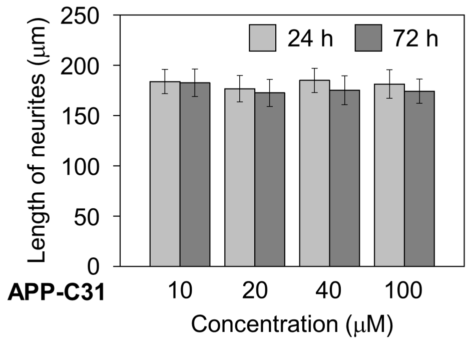

Figure S26. Analysis of the average length of neurites incubated with various concentrations of APP-C31. Conditions: [APP-C31] = 0, 10, 20, 40, and 100 μM; incubation for 24 and 72 h. Data are represented as mean ± s.e.m.; all P values obtained from Student's t-test are higher than 0.05.

References

[1] Ø. Jacobsen, J. Klaveness, O. Petter Ottersen, M. Reza Amiry-Moghaddam, P. Rongved, *Org. Biomol. Chem.* **2009**, *7*, 1599-1611.

[2] C. Toniolo, A. Polese, F. Formaggio, M. Crisma, J. Kamphuis, *J. Am. Chem. Soc.* **1996**, *118*, 2744-2745.

[3] N. A. Baker, D. Sept, S. Joseph, M. J. Holst, J. A. McCammon, *Proc. Natl. Aca. Sci. U. S. A.* **2001**, *98*, 10037-10041.

[4] T. J. Dolinsky, J. E. Nielsen, J. A. McCammon, N. A. Baker, *Nucleic Acids Res.* **2004**, *32*, W665-W667.

[5] PyMOL Molecular Graphics System by Schrödinger [http://www.pymol.org/2/ (accessed October 2019)].
